# Galectin‐3 modulates postnatal subventricular zone gliogenesis

**DOI:** 10.1002/glia.23730

**Published:** 2019-10-18

**Authors:** Osama Al‐Dalahmah, Luana Campos Soares, James Nicholson, Swip Draijer, Mayara Mundim, Victor M. Lu, Bin Sun, Teadora Tyler, István Adorján, Eric O'Neill, Francis G. Szele

**Affiliations:** ^1^ Department of Physiology, Anatomy and Genetics University of Oxford Oxford UK; ^2^ Department of Pathology and Cell Biology Columbia University Medical Center New York New York; ^3^ Department of Oncology University of Oxford Oxford UK; ^4^ Department of Anatomy, Histology and Embryology Semmelweis University Budapest Hungary

**Keywords:** electroporation, galectin‐3, gliogenesis, subventricular zone

## Abstract

Postnatal subventricular zone (SVZ) neural stem cells generate forebrain glia, namely astrocytes and oligodendrocytes. The cues necessary for this process are unclear, despite this phase of brain development being pivotal in forebrain gliogenesis. Galectin‐3 (Gal‐3) is increased in multiple brain pathologies and thereby regulates astrocyte proliferation and inflammation in injury. To study the function of Gal‐3 in inflammation and gliogenesis, we carried out functional studies in mouse. We overexpressed Gal‐3 with electroporation and using immunohistochemistry surprisingly found no inflammation in the healthy postnatal SVZ. This allowed investigation of inflammation‐independent effects of Gal‐3 on gliogenesis. Loss of Gal‐3 function via knockdown or conditional knockout reduced gliogenesis, whereas Gal‐3 overexpression increased it. Gal‐3 overexpression also increased the percentage of striatal astrocytes generated by the SVZ but decreased the percentage of oligodendrocytes. These novel findings were further elaborated with multiple analyses demonstrating that Gal‐3 binds to the bone morphogenetic protein receptor one alpha (BMPR1α) and increases bone morphogenetic protein (BMP) signaling. Conditional knockout of BMPR1α abolished the effect of Gal‐3 overexpression on gliogenesis. Gain‐of‐function of Gal‐3 is relevant in pathological conditions involving the human forebrain, which is particularly vulnerable to hypoxia/ischemia during perinatal gliogenesis. Hypoxic/ischemic injury induces astrogliosis, inflammation and cell death. We show that Gal‐3 immunoreactivity was increased in the perinatal human SVZ and striatum after hypoxia/ischemia. Our findings thus show a novel inflammation‐independent function for Gal‐3; it is necessary for gliogenesis and when increased in expression can induce astrogenesis via BMP signaling.

## Main points

- Gal‐3 loss decreased SVZ‐striatal gliogenesis, whereas overexpression increased it.
- Gal‐3 bound BMPr1‐α, increased BMP signaling and influenced glial fate choice.
- Gal‐3 was increased in human perinatal hypoxia/ischemia.

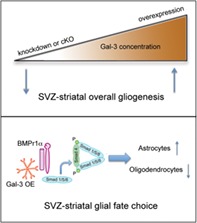

## 1 INTRODUCTION

Neural stem cells (NSCs) in the postnatal subventricular zone (pSVZ) give rise to neurogenic and gliogenic progenitors that produce olfactory bulb interneurons and forebrain glia, respectively (Levison & Goldman, 1993; Lois & Alvarez‐Buylla, 1994). Postnatal glial progenitors initially generate more astrocytes than oligodendrocytes, but this balance shifts in favor of the latter cells with increasing developmental age (Levison, Chuang, Abramson, & Goldman, 1993). Both pSVZ‐derived astrocytes and oligodendrocytes populate forebrain parenchyma and white matter, and cells originating from the lateral pSVZ settle in the striatum (Tsai et al., 2012). Yet, the signaling cues that regulate rates of postnatal SVZ gliogenesis and fate choices are not fully understood.

Galectin‐3 (Gal‐3) binds N‐glycans on glycoproteins and glycolipids, forming cross‐linking oligomers (reviewed in [Dumic, Dabelic, & Flogel, 2006; Liu & Rabinovich, 2005]). Gal‐3 is mostly undetectable in the healthy brain and has few known constitutive roles. However, we showed it is uniquely expressed in the adult SVZ, even in the absence of injury, where it regulates neurogenesis by stimulating neuroblast migration (Comte et al., 2011). Gal‐3 is usually upregulated in brain injury, inflammation and cancer (Liu & Rabinovich, 2005), promoting parenchymal astrocytic proliferation, microglial activation, angiogenesis, and immune cell influx into the SVZ (James et al., 2016; Lalancette‐Hebert et al., 2012; Sirko et al., 2015; C. C. Young et al., 2014). Other evidence has suggested Gal‐3 regulates oligodendrocyte differentiation in the adult (Pasquini et al., 2011). Disentangling constitutive effects of Gal‐3 from those of accompanying injury and inflammation has seldom been attempted. Here we found that artificially increasing SVZ Gal‐3 expression in healthy mice did not induce inflammation. Thus, we were able to distinguish Gal‐3 functions in the pSVZ from those associated with inflammation and injury, and found that it is sufficient to stimulate pSVZ gliogenesis.

Molecular studies have shown that Gal‐3 regulates several important developmental signaling pathways such as those mediated by the wingless‐related integration site (Wnt), Notch and epidermal growth factor receptor (EGFR) (Nakajima et al., 2014; Partridge et al., 2004; Song et al., 2009), but its effect on other pathways such as bone morphogenetic protein (BMP) signaling was unknown. BMP signaling peaks early postnatally and declines in adulthood (Mehler, Mabie, Zhang, & Kessler, 1997). BMP receptor activation inhibits proliferation and suppresses SVZ neurogenesis while promoting gliogenesis (Lim et al., 2000). Several groups have further shown that BMP suppresses oligodendrogenesis while promoting astrogenesis (Gomes, Mehler, & Kessler, 2003; Mabie et al., 1997; Morell, Tsan, & O'Shea, 2014). Other data suggest BMP suppresses the oligodendrocytic fate but has a permissive effect on olfactory bulb neurogenesis (Colak et al., 2008). Since Gal‐3 binds to multiple targets, influences gliogenesis in vitro and is expressed in the SVZ (Comte et al., 2011; Liu & Rabinovich, 2005) we reasoned it may regulate BMP signaling.

Human perinatal hypoxic/ischemic injury (H/I) is relatively common (Rivkin & Volpe, 1993) and can result in devastating consequences such as cerebral palsy. Periventricular regions are particularly vulnerable to H/I and the SVZ stem cell niche, which lines the lateral ventricles is likely involved in H/I‐induced abnormal gliogenesis. Understanding molecular events caused by H/I is necessary for developing therapeutic approaches, which are currently limited. A central problem in understanding molecular signals in human perinatal H/I is the scarcity of tissue specimens from postmortem neonates with H/I and the difficulty in obtaining them. Well‐matched control samples are even more rare. Another major hurdle in understanding H/I‐induced molecular cascades is the complex milieu of secondary inflammatory mediators and cellular debris that accompany cell death (Oyarce & Iturriaga, 2018). These confounding factors make it difficult to ascertain whether such molecular events are secondary to hypoxia, subsequent inflammation, or both. Here, we found that Gal‐3 is increased in the periventricular SVZ and striatum (caudate nucleus) in postmortem brain sections from human neonates with H/I.

In this study, we specifically focused on the postnatal lateral SVZ (plSVZ) since it comprises the majority of the niche. In addition, the dorsal, lateral, and ventral SVZ can harbor distinct signaling pathways and lineages and therefore should be studied separately (Azim et al., 2014; Fiorelli, Azim, Fischer, & Raineteau, 2015). We decreased and increased Gal‐3 expression via in vivo electroporation and in vitro nucleofection and interrogated BMP signaling. We found that Gal‐3 binds to the bone morphogenetic protein receptor one alpha (BMPR1α), activates BMP signaling in the plSVZ and influences the balance of plSVZ astrogenesis versus oligodendrogenesis. Using transgenic BMPR1α^fl/fl^ mice, we showed that Gal‐3 mediates its gliogenic effects through BMPR1α signaling. Finally, we demonstrated that Gal‐3 is increased in human perinatal H/I‐induced brain injury. Overall, our work demonstrates novel roles for Gal‐3 in regulating postnatal SVZ striatal gliogenesis.

## 2 MATERIALS AND METHODS

### 2.1 Animals

C57BL/6‐Tg (BMPR1α‐fx, R26R‐YFP) mice, referred to as BMPR1α^fl/fl^ were a kind gift from Dr. Tristan Rodriguez and are described elsewhere (Mishina, Hanks, Miura, Tallquist, & Behringer, 2002; Srinivas et al., 2001). C57BL/6, Gal‐3^fl/fl^, or BMPR1α^fl/fl^ mice were used for in vivo electroporation, and CD1 or C57BL/6 mice for in vitro neurosphere culture, nucleofection, western blotting, and protein co‐immunoprecipitation. Both CD1 and C57BL/6 mice were purchased from Harlan UK (Oxon, United Kingdom). Gal‐3^fl/fl^ mice (B6NTac; B6N‐Lgals3tm1a[EUCOMM]Wtsi/H), were obtained from the International Mouse Phenotyping Consortium/MRC Harwell. All animal procedures were carried out with Oxford University Research Ethics Committee approval in accordance with the Animals (Scientific Procedures) Act of 1986 (UK).

### 2.2 Human subjects

To study the effect of H/I on Gal‐3 expression in the SVZ and striatum, we selected subjects with minimal hypoxia (*n* = 2) from a former study (Adorjan et al., 2019) and subjects with more pronounced H/I (*n* = 12) from the Oxford Brain Bank (OBB) (Table S1). A further *n* = 7 subjects were selected from the OBB for study of the cerebral cortex. All human material was collected from donors from whom written informed consent had been obtained by the OBB for brain autopsy and use of material and clinical information for research purposes. Based on neuropathological analysis of hypoxic insults in the CNS and information on clinical history we stratified the perinatal cohort into four hypoxia groups with different duration of hypoxia (minimal<1 day, acute 1–2 days, subacute 3–4 days and chronic >4 days). The demographic characteristics of the cohort are shown in Table S1. Prenatal ages were described using gestational weeks (last menstruation before pregnancy).

### 2.3 Plasmids and cloning

pCAGIG (pCAG‐IRES‐GFP) was a gift from Dr. Connie Cepko (Addgene plasmid # 11159) (Matsuda & Cepko, 2004). pCAG‐Cre‐IRES2‐GFP (Addgene plasmid # 26646) (Woodhead, Mutch, Olson, & Chenn, 2006) and pTOP‐dGFP‐CAG‐mCherry (Mutch, Funatsu, Monuki, & Chenn, 2009) were gifts from Dr. Anjen Chenn. pGL3‐BRE‐Luciferase was a gift from Dr. Martine Roussel and Dr. Peter ten Dijke (Addgene plasmid # 45126) (Korchynskyi & ten Dijke, 2002). pGL4.75 (hRluc/CMV) plasmid (GenBank: AY738231, Promega) was a gift from Dr. Ian Tomlinson. pSilencer 2.0‐U6 (Ambion CAT #AM7209) containing a non‐targeting sequence (shNT) was a gift from Dr. Jo Begbie. pCS‐TdTomato‐m2A was a gift for Dr. Shankar Srinivas. Gal‐3 cDNA (NM_010705) was PCR amplified from SVZ‐derived cDNA, and Sanger sequencing confirmed the sequence. All SNP's were found to be synonymous. The sequence was cloned into pCAGIG to give rise to pCAG‐Gal‐3‐IRES‐GFP plasmid. The plasmid was digested to remove the IRES site and GFP and then ligated to give rise to pCAG‐Gal‐3 plasmid. Validated Gal‐3 short‐hairpin sequences (Henderson et al., 2006) were cloned into pSilencer 2.0‐U6 vector to produce 4 shGal‐3 plasmids. The plasmids were tested in vitro and in vivo for knockdown efficiency, and the most efficient sequence; GATGTTGCCTTCCACTTTA, was used for subsequent experiments.

### 2.4 In vivo brain electroporation

Electroporation was performed as in (Boutin, Diestel, Desoeuvre, Tiveron, & Cremer, 2008; Sun, Chang, Gerhartl, & Szele, 2018). Briefly, P2 pups were anesthetized by hypothermia. Then, 1–2 μl of plasmid(s) solution (2 μg/μl per plasmid with 0.1% Fast Green in Endotoxin‐free TE, Qiagen) was injected into the right lateral ventricle of C57BL6 or Gal‐3^fl/fl^ or BMPR1α^fl/fl^ mice. Electroporation was carried out with five 50‐ms 100 V pulses with 850 ms intervals, using CUY650‐P5 tweezers (Sonidel) connected to an ECM830 square wave electroporator (BTX). Pups recovered in a 36°C heating chamber for 15–20 min and then returned to the dam. Mice were perfused 3, 7, or 17 DPE. The electroporation efficiency was consistent and reproducible between animals, and we found that 7.8 ± 1.9% of DAPI+ SVZ cells were electroporated, N = 3, 3DPE.

### 2.5 Thymidine analog injection

BrdU (Sigma Aldrich) and EdU (Life Technologies) were reconstituted in sterile normal saline at 10 mg/ml. A single intraperitoneal (i.p.) injection of BrdU or EdU (50 mg/kg) was given.

### 2.6 Histology and fluorescent immunohistochemistry

Mice were perfused with normal saline then 4% paraformaldehyde (PFA), brains extracted, postfixed in 4% PFA, cryoprotected in 30% sucrose, frozen, and sectioned in the coronal plane on a sliding microtome. We used standard free‐floating immunohistochemistry. Briefly, 5–6 sections/animal were washed with 0.1 M PBS 3 × 10 min, incubated in 50 mM glycine in PBS for 15 min, washed with 0.1 M PBS 3 × 10 min, blocked for 1 hr in PBS + 10% Donkey Serum+0.1% Triton X‐100, before primary antibody (Ab) incubation overnight at 4°C. Three PBS washes preceded and followed incubation in secondary Ab for 1 hr at RT. DAPI (Sigma) was used to stain nuclei before section mounting and coverslipping. For BrdU and p27Kip1 detection initial an antigen retrieval step (2 N HCl for 1 hr at 37°C) was used. Human histology: coronal sections (6 μm thick) were cut from paraffin‐embedded blocks and mounted on slides. Immunohistochemical analysis was done as described in detail (Adorjan et al., 2017). Briefly, anti‐Galectin‐3 antibody (Abcam, ab 2,785, 1:200, mouse, monoclonal) was applied following heat‐induced antigen retrieval in a 121°C autoclave for 10 min in EDTA (pH = 9.0) and revealed with 3,3′‐Diaminobenzidine (DAB).

The following primary Ab's were used: chicken α‐GFP (1:500; Avislabs), rabbit α‐Mcherry (1:750; Abcam), rabbit α‐Ki67 (1:500; Abcam), rabbit α‐S100β (1:200; Dako), mouse α‐vimentin (1:100; DSHB), rabbit α‐Caspase‐3 (1:500; Cell Signaling), rabbit α‐Smad 1/5/8 (1:500; Cell Signaling), rabbit α‐pSmad1/5 (Ser463/465) (1:500; Cell Signaling), rat α‐GFAP (1:500; Invitrogen), goat α‐Dcx (1:100; Santa‐Cruz), rat α‐Galectin‐3 (1:100; Santa‐Cruz), rat α‐BrdU (1:600; Novus Biologicals), rat α‐CD45 (1:400; Millipore), rabbit α‐Olig2 (1:1000; Millipore), mouse α‐NeuN (1:400; Millipore), goat α‐Iba1 (1,300; Abcam), rabbit α‐β‐catenin (1,1000; Sigma), mouse α‐Mash1 (Ascl1) (1,100; BD Biosciences). Secondary antibodies conjugated to Alexa Fluor 488, 568, 594, or 647 (Invitrogen, Sigma and Jackson Laboratory) were used as appropriate. For EdU detection, a Click‐iT® EdU detection kit (Invitrogen) was used and the manufacturer's protocol adapted to free‐floating IHC. Standard LacZ histochemistry was carried out (Jackson Laboratory website).

### 2.7 Image acquisition and quantification

Confocal images were acquired on a Zeiss LSM 710 microscope. All images represent single optical planes, unless noted. All quantifications were done by an observer blinded to experimental condition. For co‐localization studies, 40X *Z*‐stacks and 20X *Z*‐stacks (12 optical sections, each 1.3 μm apart) were quantified with Volocity 6.3 (Improvision) and ImageJ. At least three images from three sections were quantified per animal and considered technical replicates. The sections spanned the lateral ventricle (LV) at the level of, and anterior to, the crossing of the anterior commissure. Quantifications were done in the lateral SVZ only, and the dorsolateral horn of the SVZ was excluded. Only DAPI+ cells were included, and cells the nuclei of which were not completely within the z‐stack were excluded.

### 2.8 Protein extraction, immunoprecipitation, and western blotting

Dissected SVZ tissue was lysed in Triton X‐100 lysis buffer (1% Triton‐X‐100, 150 mM NaCl, 50 mM Tris HCl pH 8.0, Roche Complete protease inhibitor cocktail). HEK293T cells were seeded into 6‐well plates (200,000 cells/well) in 2 ml of growth media (DMEM +10% Fetal Bovine Serum +100 I.U/ml Penicillin G and 100 mg/ml Streptomycin) 24 hr before transfection. The following day, cells achieved 70% confluence, which is suitable for transfection. One hour prior to transfection, the media was changed to Opti‐MEM® (Life Technologies). Then, 2.0 μg of plasmid DNA (pCAG‐Gal‐3‐IRES‐GFP and pCMV3‐SP‐HA‐BMPR2 (Sino Biological) and/or pCMV3‐mBMPR1α‐Flag (Sino Biological)) was used with Lipofectamine 3,000™ (Life Technologies) for transfection according to manufacturer's protocol. The media was changed to growth media with 20% FBS 12 hr post transfection, and transfection efficiency (70–80%) was verified using GFP fluorescence. The cells were harvested for protein 48 hr post‐transfection. Immunoprecipitation (IP) was performed using μMACS™ protein A/G microBead magnetic separation (Miltenyi Biotec 130–071‐001) according to the manufacturer's manual. Briefly, 200 μg of protein, 1 μg of monoclonal antibody (mouse anti‐Octa probe (H5); Santa Cruz sc‐166355, mouse anti‐HA F‐7; Santa Cruz sc‐7392, rat anti‐Gal‐3 (M3/38); Santa Cruz sc‐23938, or 2 μg of control polyclonal antibody, and 50 μl of protein G microbeads (Miltenyi Biotec) were mixed by vortexing and incubated for 30 min on ice. The magnetically labeled lysate was passed through a MACS μcolumn placed in a magnetic field. The columns were washed and the elution followed. The eluent protein was then electrophoresed on NuPage BT 4–12% gels for western blot analysis. Immunoblotting for Gal‐3 (rabbit anti‐Gal‐3 [H‐160]; Santa Cruz sc‐20157) FLAG (mouse anti‐Octa probe (H5); Santa Cruz sc‐166355, or rabbit anti‐DYKDDDDK Tag; CS‐2368), or BMPR1α (Rabbit anti‐BMPR1α, Invitrogen 38‐6000) was followed by visualization on a LiCOR Odyssey system after blotting with appropriate IRDye‐fluorescent secondary antibodies.

### 2.9 Statistical analyses

All mouse quantification was done blind to experimental condition using a random coding system. Differences between two groups were assessed using unpaired *t*‐test when normality could be checked using Shapiro–Wilk test (*n* = 5 or more) or the Mann–Whitney U nonparametric test (*n* = 3 or 4). For in vitro experiments, including qPCR analysis of delta–delta Ct values from biological replicates, one sample *t*‐test was used after data normalization to control. Comparisons between three or more groups were conducted using analysis of variance (ANOVA) with Tukey or Sidak post hoc tests as appropriate in experiments with *n* ≥ 5, otherwise, the Kruskal Wallis test was used, unless otherwise indicated. For analysis of human Gal‐3+ cells, a one tailed *t*‐test (samples with unequal variance) was used in Excel to compare the percentage of strongly Gal‐3+ cells among all cells counted by a standardized algorithm (Supporting materials and methods). Significance is indicated as follows: *p < .05, **p < .01, ***p < .001, ****p < .0001. Values were presented as mean ± *SEM*, unless otherwise indicated. Analyses and graphical representations were performed in Microsoft Excel 2011 and GraphPad Prism 6 (GraphPad) software packages. All other methods are described in Supplementary Materials and methods.

## 3 RESULTS

### 3.1 Galectin‐3 does not induce inflammation or apoptosis in the postnatal SVZ

We have previously shown that most SVZ cells except for neuroblasts constitutively express Gal‐3 in the postnatal and adult niche (Comte et al., 2011; Hillis, Davies, Mundim, Al‐Dalahmah, & Szele, 2016; James et al., 2016; C. C. Young et al., 2014). We found the same pattern of expression in the pSVZ in this study (Figure S1). We used immunohistochemistry for glial fibrillary acidic protein (GFAP) to label SVZ astrocytes (a subset of which are NSCs), Mash1 (Ascl1) to label transit amplifying progenitors, and doublecortin (Dcx) to label neuroblasts (Figure S1). Gal‐3 was expressed by a subset of GFAP+ and Mash1+ SVZ cells but was not detectable in Dcx+ neuroblasts.

We next validated Gal‐3 knockdown and overexpression constructs in vitro and in vivo (Figure S2a). Three days after transfection of HEK293T cells, western blotting was used to compare Gal‐3 protein levels between different combinations of GFP expressing control plasmids, Gal‐3 overexpression, Gal‐3 knockdown and non‐targeting shRNA (shNT) control. The OE plasmid increased Gal‐3 levels from baseline and 4 shGal‐3 plasmids (V1‐4) knocked down Gal‐3 after OE (baseline levels of Gal‐3 were very low in HEK293T cells, Figure S2a). V1 was most efficient (Figure S2a) and we used it throughout the study unless otherwise specified. We next examined the effects on Gal‐3 expression of these constructs in vivo by co‐electroporating them with a plasmid expressing mCherry into the plSVZ of P2 mice, as in (Sun et al., 2018). Quantitative comparison of Gal‐3 immunofluorescence with mCherry showed that already at 3 days post electroporation (3DPE) of shGal‐3, Gal‐3 immunofluorescence was significantly decreased in SVZ cells (Figure S2b,c). Conversely, Gal‐3 OE significantly increased Gal‐3 immunofluorescence (Figure S2b,c). Gal‐3 knockdown was next assessed in vivo at 17DPE with co‐electroporation of a tdTomato expressing plasmid. The percentage of tdTomato positive cells that exhibited Gal‐3 immunofluorescence was significantly reduced by the V1 and V2 shGal3 constructs (Figure S2d,e).

**Figure 1:**
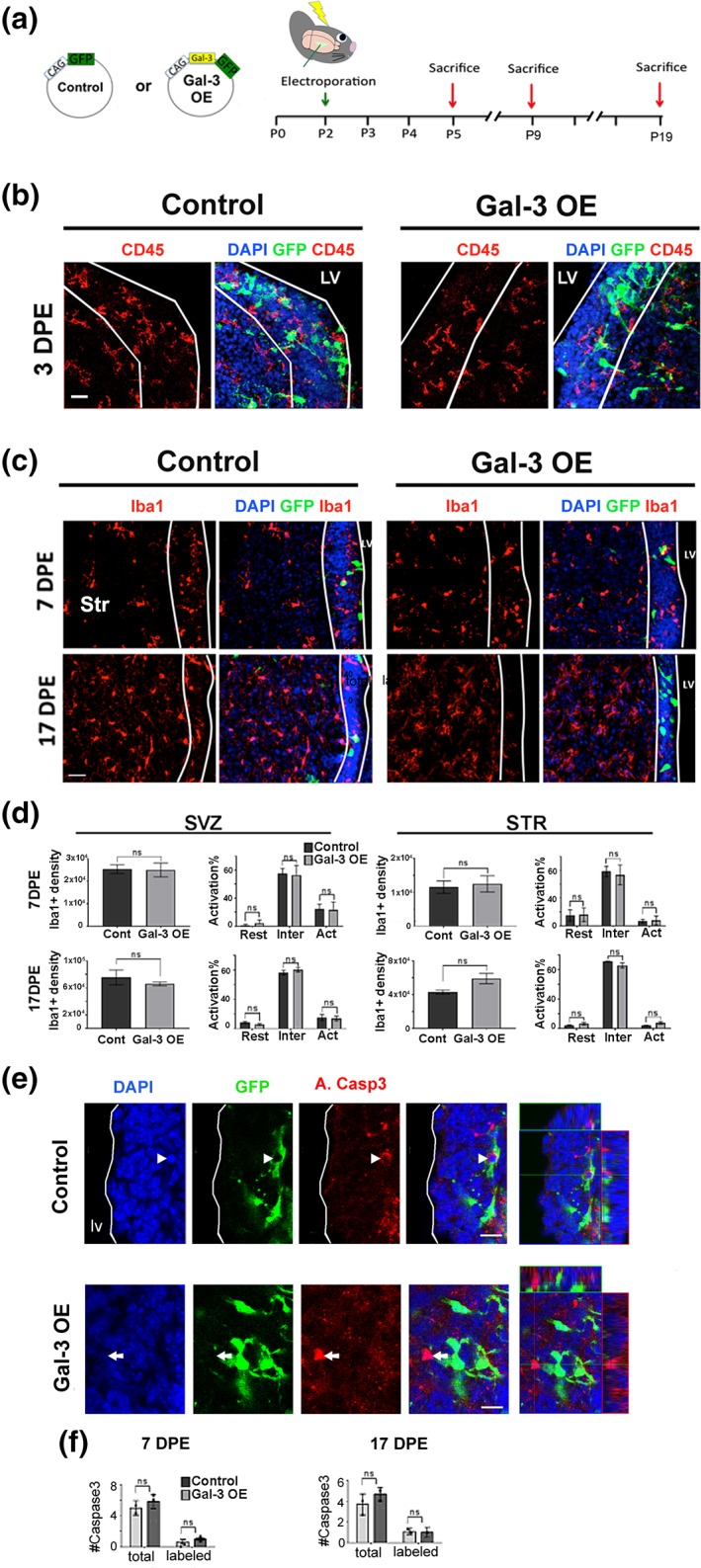
Gal‐3 did not induce inflammation in the absence of injury. (a) Plasmids used and timeline of in vivo brain electroporation. (b) Representative confocal collapsed stacks of control and Gal‐3 OE 3DPE showing DAPI+ nuclei (blue), electroporated GFP+ cells (green), and CD45+ immunohistochemistry (red). LV, lateral ventricle. Scale bar: 20 μm. (c) Representative sections stained for Iba1 and DAPI, 7 and 17DPE. The SVZ is delineated with white lines. STR, striatum. Scale bar: 30 μm. (d) Quantification of Iba1+ microglial density/mm^3^ and activation status as resting (Rest), intermediate (Inter), activated (Act) in the SVZ, and striatum (STR). *n* = 3–4. (e) Representative confocal images at 17DPE; electroporated GFP+ cells (green), A.Casp3 (red), and DAPI+ nuclei (blue) from control and Gal‐3 OE. The arrow indicates a GFP+ A.Casp3+ cell, whereas the arrowhead indicates a GFP‐A.Casp3+ cell. Orthogonal views of the indicated cells are shown on the left. Scale bars: 10 μm. (f) Quantification of the mean number of Caspase3+ cells per animal at 7 and 17 DPE. Statistical significance was determined using Mann–Whitney test. Data are shown as mean ± *SEM*. *p < .05. Gal‐3, Galectin‐3; ns, nonsignificant; SVZ, subventricular zone

Inflammation and injury are associated with elevated Gal‐3 (James et al., 2016; Y. Li et al., 2008; Lopez et al., 2006) and inflammation affects gliogenesis, neurogenesis, and proliferation in neural stem cell niches (Ekdahl, Claasen, Bonde, Kokaia, & Lindvall, 2003; Monje, Toda, & Palmer, 2003; Ribeiro Xavier, Kress, Goldman, Lacerda de Menezes, & Nedergaard, 2015; Shigemoto‐Mogami, Hoshikawa, Goldman, Sekino, & Sato, 2014). Since Gal‐3 modulates inflammation and levels of Gal‐3 are increased in inflammatory conditions, it is difficult to distinguish if effects of increased Gal‐3 are primary or secondary to inflammation. To study Gal‐3's effect on plSVZ cellular inflammation, we used antibodies against CD45, a pan‐immune cell marker (Goings, Kozlowski, & Szele, 2006). We qualitatively assessed CD45+ cells blind to the treatment and found that microglial morphology and numbers were unchanged in the plSVZ upon Gal‐3 OE at 3, 7, and 17DPE (Figures 1a,b and S3a). Iba1 immunohistochemistry was next used to examine microglial cells that were potentially activated by Gal‐3 OE. Analysis at 3, 7, and 17 DPE showed that Iba1+ cell density per volume was unchanged by Gal‐3 OE in the SVZ as well as in the striatum (Figures 1c,d and S3b,c). A detailed morphological analysis of Iba1+ microglia revealed that most were in an intermediate state of activation at all three ages examined (Figure S3c,d). Gal‐3 OE also did not change the level of microglial morphological activation in the SVZ or in the striatum at 3, 7, and 17DPE (Figures 1d and S3c,d).

Inflammation can cause apoptosis, thus we examined a marker of apoptosis, activated Caspase‐3+ (Alfonso‐Loeches, Pascual‐Lucas, Blanco, Sanchez‐Vera, & Guerri, 2010). Gal‐3 OE did not change the number of electroporated SVZ cells expressing activated Caspase‐3 at 7 or 17DPE (Figure 1e,f). Similarly, quantification showed that the number of Caspase‐3 cells in the OB 7DPE was unchanged in the OE group compared to controls (Figure S3e). Moreover, we found no evidence of an increase in electroporated cells that co‐labeled with A.Caspase 3 or A.Caspase 3 and Olig2 at 3DPE (Data not shown). This suggests that Gal‐3 did not affect programmed cell death, that Gal‐3 OE did not elicit an inflammatory response in the plSVZ, and that Gal‐3 effects on the SVZ are independent of microglial inflammation.

### 3.2 Gal‐3 is necessary and sufficient for striatal gliogenesis

**Figure 2:**
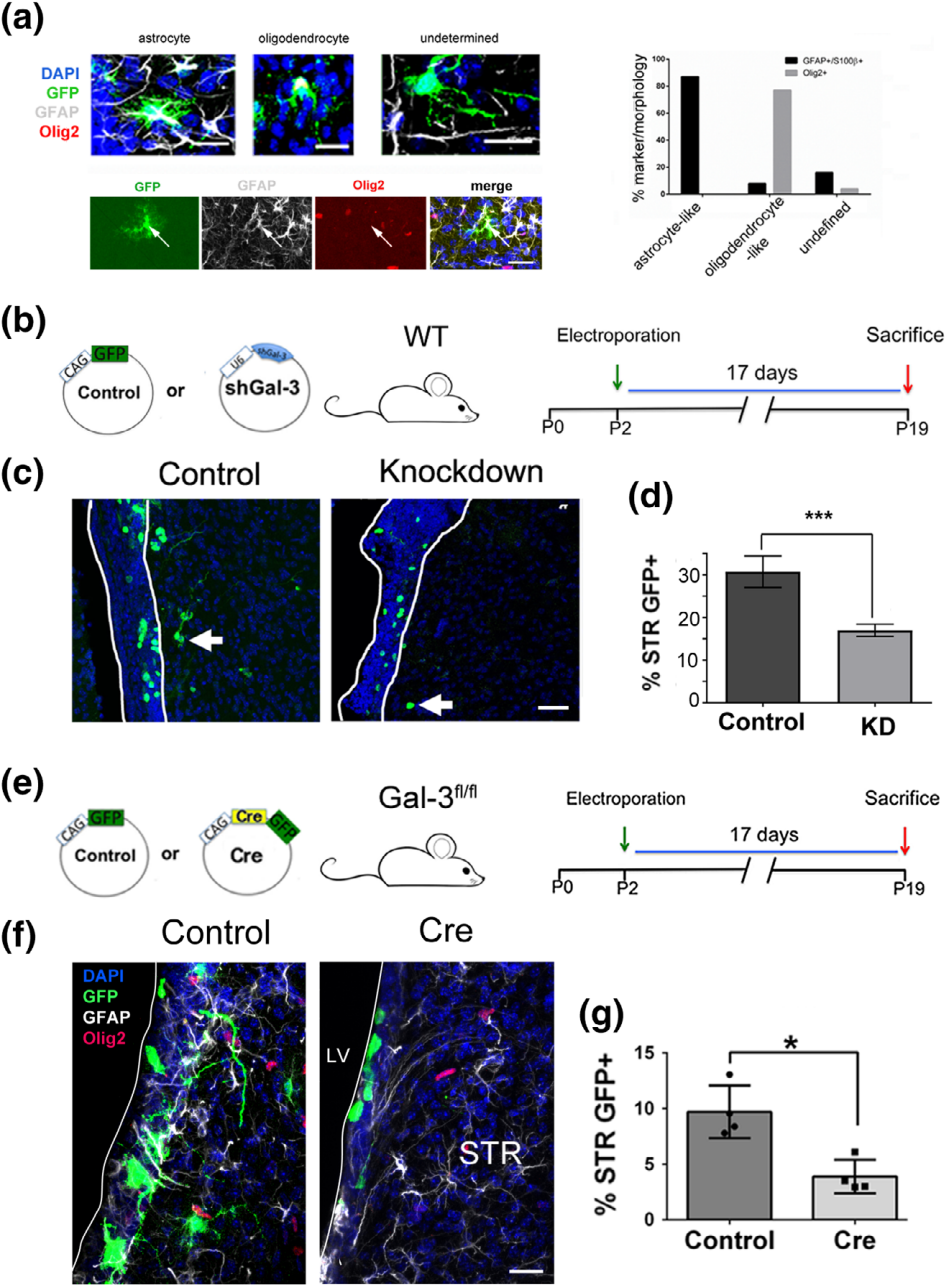
Gal‐3 loss‐of‐function regulates gliogenesis. (a) The morphological criteria used to classify GFP+ cells as oligodendrocytes or astrocytes largely matched marker expression. The top left panel shows a GFAP+ astrocyte‐like cell, an Olig2+ oligodendrocyte‐like cell and an undetermined cell. Note that GFP and glial marker double labeling appears white in the merged images. Bottom left panel shows example of a GFP + GFAP+ astrocyte (arrow) that is negative for Olig2. Scale bars = 30 μm. (b) Plasmids, mice, and design of Gal‐3 knockdown in vivo loss‐of‐function electroporation. (c) Representative confocal images of GFP+ cells 17DPE of control and knockdown constructs. The white lines demarcate the SVZ. Scale bar: 30 μm. (d) Quantification of B as percent of SVZ + striatal GFP+ cells that are striatal. (e) Plasmids, mice, and design of Gal‐3 conditional knockout in vivo loss‐of‐function electroporation. (f) Representative confocal images of GFP+, GFAP+, Olig2+ cells, and DAPI+ nuclei. 17DPE with control or Cre‐plasmids. The white line demarcates the lateral ventricle. Scale bar: 15 μm. (g) Quantification of E as percent of SVZ + striatal GFP+ cells that are striatal. Statistical significances were calculated using Mann–Whitney *U* tests. *n* = 3–4 mice/group. Gal‐3, Galectin‐3; SVZ, subventricular zone

The large majority of postnatal SVZ progenitors that migrate into the striatum differentiate into glia, as opposed to neurons (Levison et al., 1993; Levison & Goldman, 1993). We categorized striatal GFP+ cells as astrocytes, oligodendrocytes, or others/undetermined using established morphological criteria and approximately 99% of GFP+ cells in the striatum were classified as glia (Supplementary materials and methods). Using these criteria, the large majority of cells morphologically classified as astrocytes or oligodendrocytes expressed GFAP/S100β and Olig2, respectively (Figure 2a). To study Gal‐3's effects on this process, we altered Gal‐3 levels in the SVZ at P2 and harvested brains at P19 giving sufficient time for gliogenesis (Figure 2a). To determine if Gal‐3 is necessary for striatal gliogenesis, we first electroporated Gal‐3 KD constructs into the P2 SVZ (Figure 2b,c). In order to compensate for slight variations in electroporation efficiency, we determined what percentage of total GFP+ cells (SVZ + striatum) where found in the striatum. This percentage was statistically significantly decreased after Gal‐3 knockdown indicating that Gal‐3 is necessary for gliogenesis (Figure 2d). We next confirmed the Gal‐3 knockdown effect with Gal‐3^fl/fl^ mice (Figures 2e–f and S2f–j). We first showed statistically significant loss of Gal‐3 expression in the SVZ of animals electroporated with GPF‐Cre compared to control GFP plasmids (Figure S2i,j) (17DPE, *n* = 3 mice/group, ~70 cells/mouse quantified). Compared with control GFP plasmid electroporation, Cre‐electroporated Gal‐3^fl/fl^ mice had a significantly smaller percentage of total GFP+ glial cells in the striatum at 17DPE (Figure 2f,g). Double labeling with GFAP and Olig2 antibodies confirmed that GFP+ cells in the striatum were positive for either one or the other glial marker, confirming SVZ—striatal gliogenesis (Figure 2f). These data show that endogenous Gal‐3 is overall necessary for striatal gliogenesis from the plSVZ, revealing a novel constitutive neurodevelopmental function for this protein.

**Figure 3:**
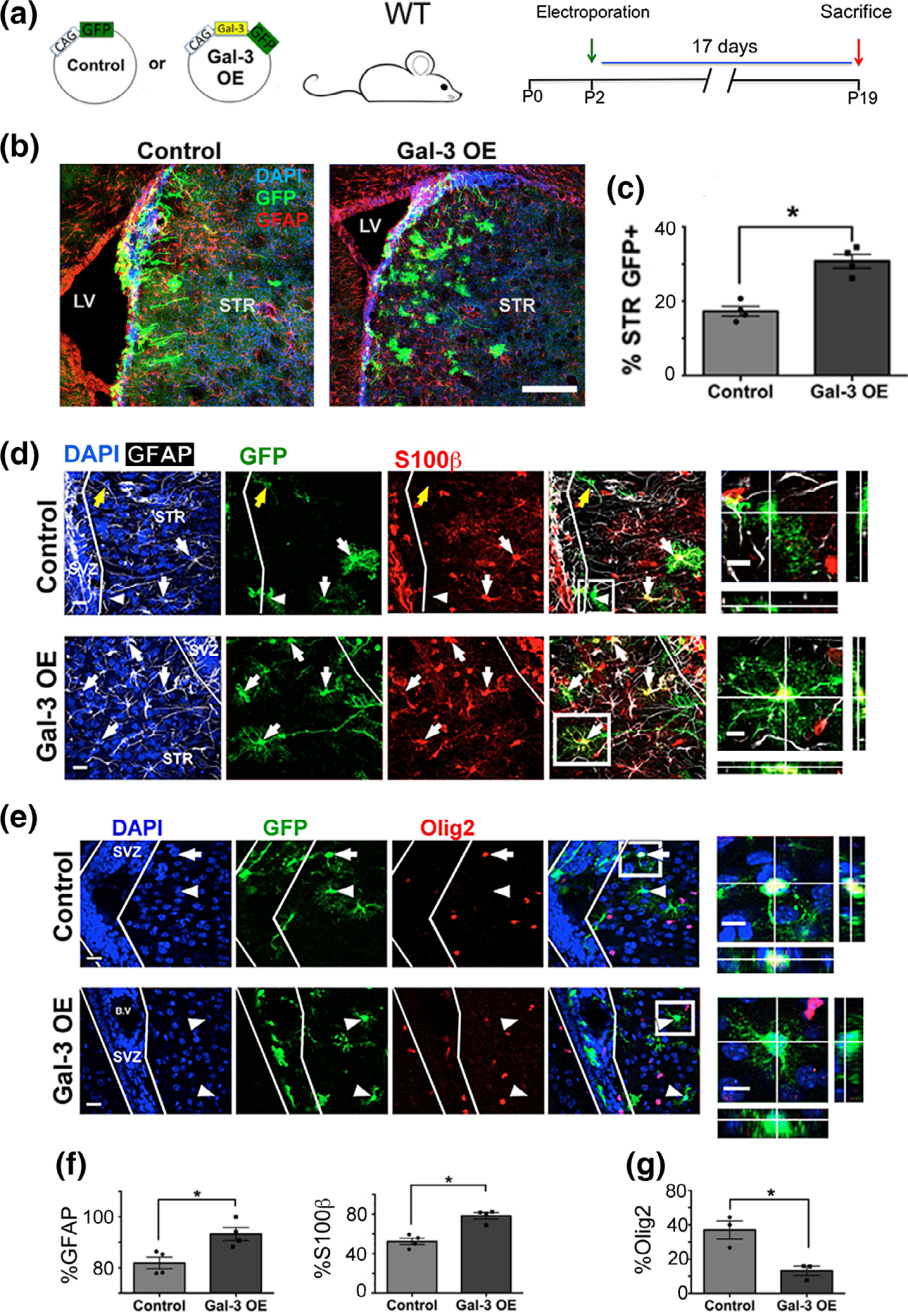
Gal‐3 gain‐of‐function regulates gliogenesis. (a) Plasmids, mice and design of Gal‐3 OE in vivo gain‐of‐function electroporation. (b) Confocal images including SVZ and striatum (STR). DAPI+ nuclei, GFP+, and GFAP+ cells shown. (c) Quantification of H as percent of SVZ + striatal GFP+ cells that are striatal. (d) Projection images showing DAPI+ nuclei, GFAP+, GFP+ and S100β + cells in controls and Gal‐3 OE. White arrows indicate GFP+GFAP+S100β + cells, the yellow arrows GFP+GFAP+S100β‐ cell, and arrowheads GFP+GFAP‐S100β− cell in the STR. (e) Single confocal planes showing DAPI+ nuclei, GFP+, and Olig2+ cells. Arrowheads point to GFP+Olig2‐ cells and the arrow to a GFP+Olig2+ cell. (d, e) Boxed areas shown as magnified and orthogonal views on the left. (f) Quantification of D as percent of striatal GFP+ cells that are GFAP+ or S100β+. G: Quantification of E as percent of striatal GFP+ cells that are Olig2+. Scale bar for B: 200 μm. Scale bars for D, E: 20 μm for main panels, 10 μm for insets. Statistical significances were calculated using Mann–Whitney U tests. *n* = 3–4 mice/group. Gal‐3, Galectin‐3; SVZ, subventricular zone

Contrary to the loss‐of‐function effects, Gal‐3 OE in WT mice significantly increased the proportion of GFP+ glial cells in the striatum at 17DPE (Figure 3a–c). Increased striatal gliogenesis occurred without alteration in the total number of electroporated cells, the rate of SVZ proliferation, the percent of astrocytes or the percent of proliferative astrocytes at 17DPE (Figure S4a–e).

We next looked at the earlier survival time of 7DPE and similar to 17DPE, Gal‐3 OE did not affect SVZ proliferation, the number of label‐retaining cells or cell cycle re‐entry (Figure S5a‐e). At 7DPE, Gal‐3 OE also did not increase the percent of GFP+ cells that expressed Ki67 (Ki67 labeling index) or the percent of GFP+ cells that expressed GFAP (Figure S5f–h). However, Gal‐3 OE did increase the number of electroporated GFP+ cells that were GFAP+Ki67+ (Ki67 GFAP+ labeling index) in the SVZ (Figure S5f,i). Ki67 labels multiple phases of the cell cycle and thus the effect could have been due to prolonged phases of the cell cycle, rather than increased proliferation. Contrary to the Ki67 labeling index, which measures the growth fraction and is influenced by changes in cell‐cycle length, the M‐phase of the cell‐cycle labeled by Phi3 is relatively stable. Thus, changes in the Phi3 labeling index would reflect changes in proliferation rather than cell‐cycle length. We therefore determined whether Gal‐3 OE increases Phi3 at 7 DPE in GFAP+GFP+ cells. We found that similar to Ki67, the percent of GFP+ cells that were Phi3+ was unchanged by Gal‐3 OE (Figure S5l). Also, similar to Ki67, the percent of GFAP+GFP+ cells that expressed Phi3 was significantly increased by Gal‐3 OE (*p* = .032 – one‐tailed *t*‐test) (Figure 5Sk). In contrast to its effects on striatal gliogenesis, we found no evidence that Gal‐3 OE has an effect on olfactory bulb neurogenesis at 17DPE (Figure S6a–e). Together, these data show that Gal‐3 OE selectively increases the Ki67 and Phi3 labeling indices (and therefore overall proliferation) of a subset of SVZ GFAP+ cells without altering olfactory bulb neurogenesis or NSC activation (Supplementary results).

We noticed after Gal‐3 OE in the SVZ that many striatal GFP+ cells had altered morphology compared to controls (Figure 3b). To determine the astrocytic identity and differentiation state of electroporated cells, we examined GFAP and S100β expression (both mature astrocyte markers, Figure 3d) and vimentin (immature astrocyte marker Figure S7a). More electroporated GFP+ cells in the striatum expressed GFAP or S100β after Gal‐3 OE (Figure 3f), and fewer expressed vimentin (Figure S7b). These data suggest that in the striatum, Gal‐3 OE increases astrocytic differentiation of SVZ‐derived glia. In contrast to GFAP and S100β, significantly fewer labeled cells expressed the oligodendrocyte marker Olig2 after Gal‐3 OE (Figure 3e,g). These data indicate that Gal‐3 OE in the plSVZ caused a shift in SVZ glial progenitor fate choices by augmenting astrogenesis and reducing oligodendrogenesis.

### 3.3 Galectin‐3 overexpression increases BMP signaling

**Figure 4:**
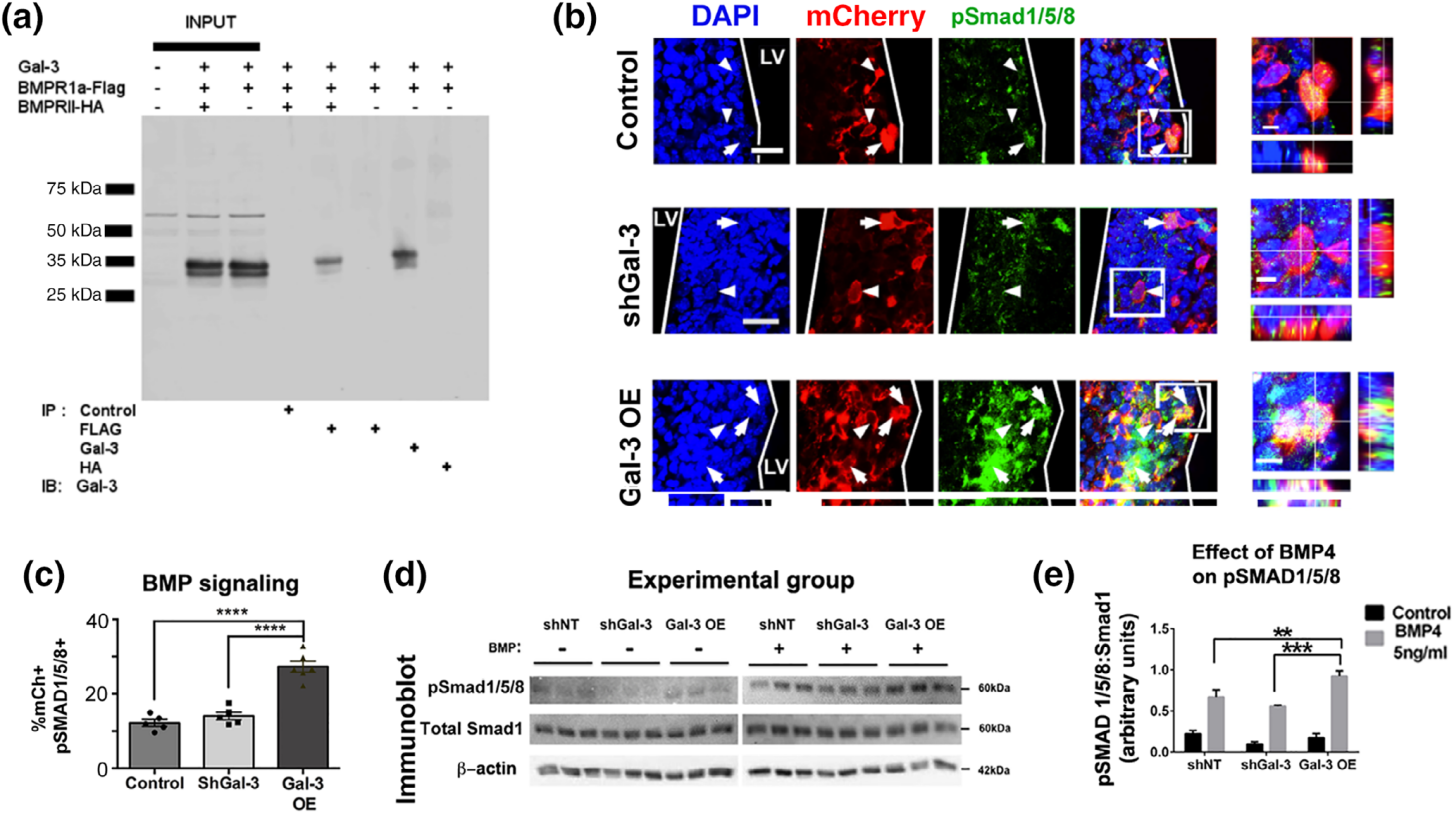
Gal‐3 increases BMP signaling. (a) Co‐immunoprecipitation of Gal‐3 and BMPR1α and BMPR2. (b) Representative confocal images 3DPE. DAPI+ nuclei, mCherry+ electroporated cells, and pSmad 1/5/8+ cells. Arrows indicate pSmad1/5/8+ and arrowheads pSmad1/5/8‐labeled cells. Boxed areas are enlarged as orthogonal views. Scale bars: 20 μm for main panels, 5 μm for insets. (c) Quantification of B. One‐way ANOVA, Tukey's test for multiple comparisons. *n* = 5–6. (d) Western blot 24 hr after nucleofection with shNT (control), shGal‐3, or Gal‐3 OE, cells were treated with control or 5 ng/ml BMP4 for 24 hr. Immunoblot for pSmad 1/5/8, total Smad1 and β‐Actin. (e) Quantification of pSmad1/5/8 band intensities from (A1‐2) normalized to total Smad 1. 2‐way ANOVA

Since BMP signaling in the SVZ reduces oligodendrogenesis, and promotes astrocyte differentiation (Gomes et al., 2003) we examined the potential role of this signaling pathway in Gal‐3 function. We first confirmed that Gal‐3 binds to BMPR1α as has been suggested (Zhang et al., 2017) and then tested the hypothesis that BMPR1α is necessary for Gal‐3's effects. We expressed Gal‐3, Flag‐tagged BMPR1α, and HA‐tagged BMPR2 in HEK293T cells and determined if Gal‐3 and BMPR1α and/or BMPR2 co‐immunoprecipitate. Gal‐3 was complexed with BMPR1α but not BMPR2 (Figure 4a). The role of Gal‐3 on BMP signaling was unknown, thus, we assessed the in vivo SVZ expression of phosphorylated‐Smad/1/5/8 (pSmad1/5/8), which is increased upon canonical BMP signaling. We confirmed that a subset of pSmad1/5/8+ cells in controls co‐expressed Gal‐3 (Figure S7c). This suggested baseline Gal‐3 may regulate BMP signaling, yet Gal‐3 knockdown did not affect pSmad1/5/8 levels in the SVZ (Figure 4b,c). Conversely, Gal‐3 OE increased pSmad1/5/8 immunofluorescence and more than doubled the percentage of pSmad1/5/8+ electroporated cells in the SVZ 3DPE (Figure 4b,c).

We next studied the effects of Gal‐3 and BMP4 on pSmad1/5/8 levels compared to total Smad5 or Smad1 using western blots. Gal‐3 regulated BMP signaling by increasing the phosphorylation of pSmad1/5/8 without altering total Smad protein content (Figures 4d,e and S7d,e). To confirm that Gal‐3 increases BMP signaling, a BMP signaling reporter (BRE‐Luciferase) was used to measure BMP signaling in plSVZ‐derived neurospheres after Gal‐3 OE or KD. Consistent with the in vivo data, BMP signaling was significantly increased 2 days after Gal‐3 OE compared to control, whereas knockdown caused slight but non‐significant reductions in BMP signaling (Figure S7f). These data show a novel function for increased Gal‐3, it binds to BMPR1α and positively regulates BMP signaling in the plSVZ.

To determine if the converse is true and if BMP signaling regulates Gal‐3 expression we used qPCR after BMP4 stimulation of neurospheres in vitro. As expected (Ying, Nichols, Chambers, & Smith, 2003), Id1 levels were increased after BMP4 treatment, providing a positive control (Figure S7g). In contrast, Gal‐3 mRNA levels were reduced 24 and 48 hr after BMP4 treatment of SVZ cells (Figure S7g), suggesting negative feedback on Gal‐3 transcription. Thus, our data indicate that while Gal‐3 induced BMP signaling, BMP signaling in turn suppressed Gal‐3 expression.

### 3.4 BMPR1α signaling is necessary for galectin‐3 to increase astrogenesis

**Figure 5:**
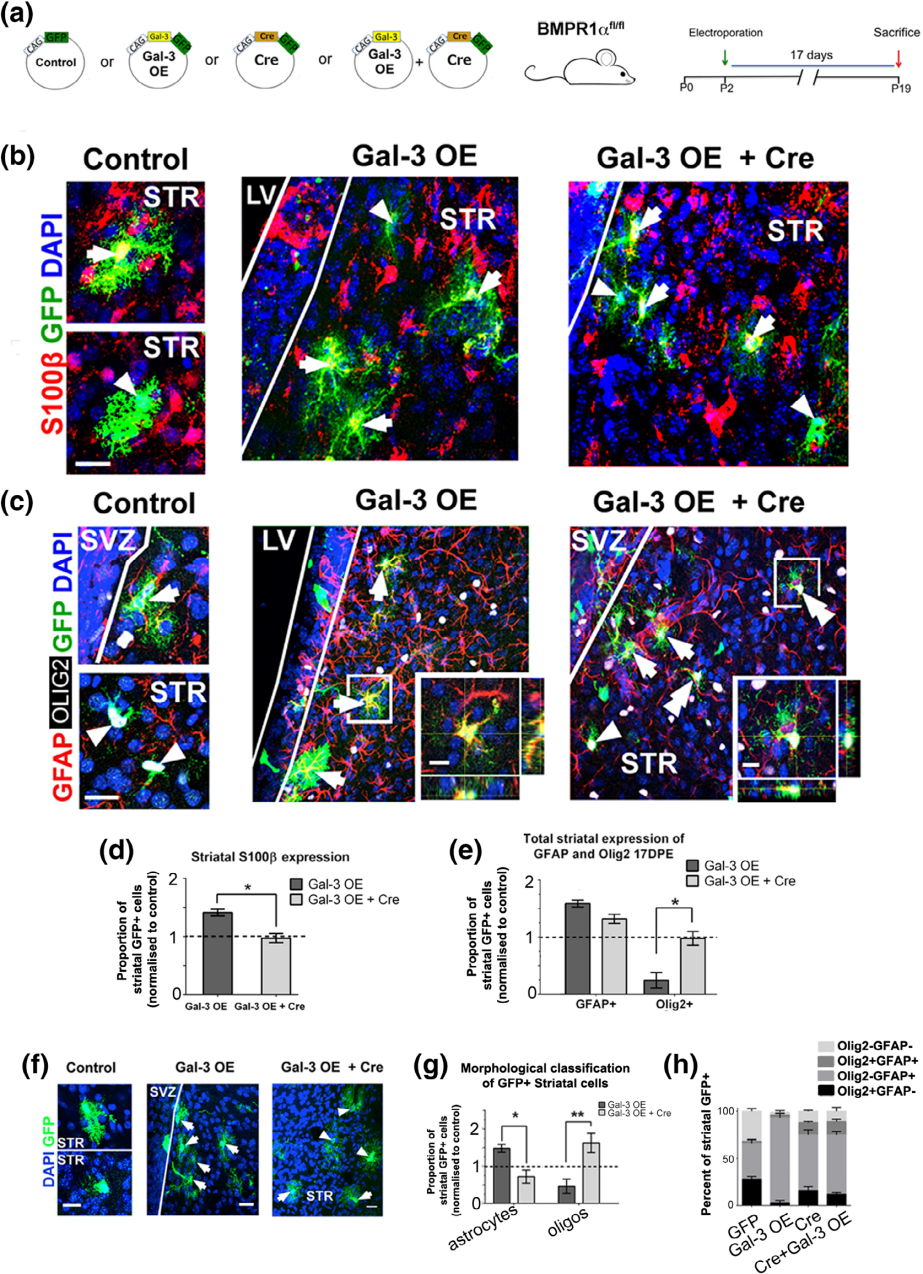
BMPR1α is necessary for Gal‐3's effects. (a) Plasmids, mouse, and electroporation. (b) Confocal images, arrows indicate S100β + and arrowheads S100β negative cells. Scale bars: 20 μm in panels,10 μm in insets. (c) Quantification of B showing GFP control‐normalized proportions of S100β + striatal cells. (d) Confocal and projection images, arrows indicate GFAP+, arrowheads Olig2+, and double arrowheads GFAP+Olig2+ cells. (e) Quantification of D. Mann–Whitney U test. *n* = 3–5. Mean ± *SEM*. *p* value: * < .05. (f) Maximal projection images showing morphological assessment of striatal cells 17DPE in controls, Gal‐3 OE, or Gal‐3 OE + Cre in BMPR1α^fl/fl^ mice. Arrows indicate astrocyte‐like cells (upper panel in the control group), and arrowheads oligodendrocyte‐like cells (lower panel in the control group). Scale bars: 20 μm. (g) The GFP control‐normalized proportion of striatal cells that were morphologically oligodendrocyte‐like increased whereas those that were astrocyte‐like decreased in the Gal‐3 OE + Cre in BMPR1α^fl/fl^ mice compared to Gal‐3 OE. Mann–Whitney U test. *n* = 3–5. (h) Co‐expression patterns of GFAP and Olig2 in electroporated groups. The percent of labeled GFP+ cells that were either Olig2+ or GFAP+ was not significantly different between the Cre and Gal‐3 OE + Cre groups. Gal‐3, Galectin‐3; SVZ, subventricular zone

We found that Gal‐3 positively regulates BMP signaling, thus we next investigated if Gal‐3's effect on SVZ gliogenic fate choice is dependent on BMP signaling. Control, Gal‐3 OE, Cre or Gal‐3 OE + Cre plasmids were electroporated into the plSVZ of P2 BMPR1α^fl/fl^ mice and brains harvested at 17DPE (Figure 5a). We used antibodies against S100β and GFAP to label astrocytes, and Olig2 to label oligodendrocytes (Figure 5b,d). Cre plasmids electroporated with Gal‐3 OE abolished the increase in the proportion of S100β + newborn glial cells observed in Gal‐3 OE alone (Figure 5c), suggesting that BMPR1α signaling was necessary for the Gal‐3 OE effects on glial fate choice. Compared to Gal‐3 OE alone, co‐electroporating Cre plasmids with Gal‐3 OE showed a non‐significant reduction in the proportion of GFP+ striatal cells that expressed GFAP (*p* = .07) (Figure 5e). However, the proportion of GFP+ striatal cells that expressed Olig2 after Gal‐3 OE was normalized (*p* < .05) from very low to control levels upon conditional loss of the BMPR1α (Figure 5e). These data indicate that loss of the BMPR1α restored oligodendrogenesis and partially rescued the increased astrogenesis after Gal‐3 OE.

To further confirm that BMPR1α was required for inducing astrogenesis after Gal‐3 OE we examined cell morphology (Figure 5f). Confirming our data above, Gal‐3 OE alone increased the percentage of cells with astrocytic morphology compared to controls but decreased the percentage of cells with oligodendrocytic morphology (Figure 5g). In contrast, co‐electroporation of Gal‐3 OE with Cre to remove BMPR1α reversed this effect and significantly increased oligodendrocyte‐like cells (*p* < .01) and decreased astrocyte‐like cells (*p* < .05) (Figure 5g). Electroporation of Cre plasmids only were indistinguishable from Cre + Gal‐3 OE, providing further evidence that signaling through BMPR1α is downstream to Gal‐3's action. Interestingly, we found that co‐electroporating Cre + Gal‐3 OE significantly increased the population of double‐positive GFAP+Olig2+ cells in the striatum (2.08 ± 2.95% control, 3.14 ± 2.78% Gal‐3 OE; 14.47 ± 2.69% Gal‐3 OE + Cre; *p* < .01 one‐way ANOVA, Figure 5h). This suggests that loss of BMP signaling de‐repressed Olig2+ expression in GFAP+ striatal glia. Taken together, we provide strong evidence that Gal‐3's effect on gliogenic fate choice is dependent on BMP signaling.

### 3.5 Galectin‐3 expression increases in the forebrain of patients with perinatal hypoxia ischemia

**Figure 6:**
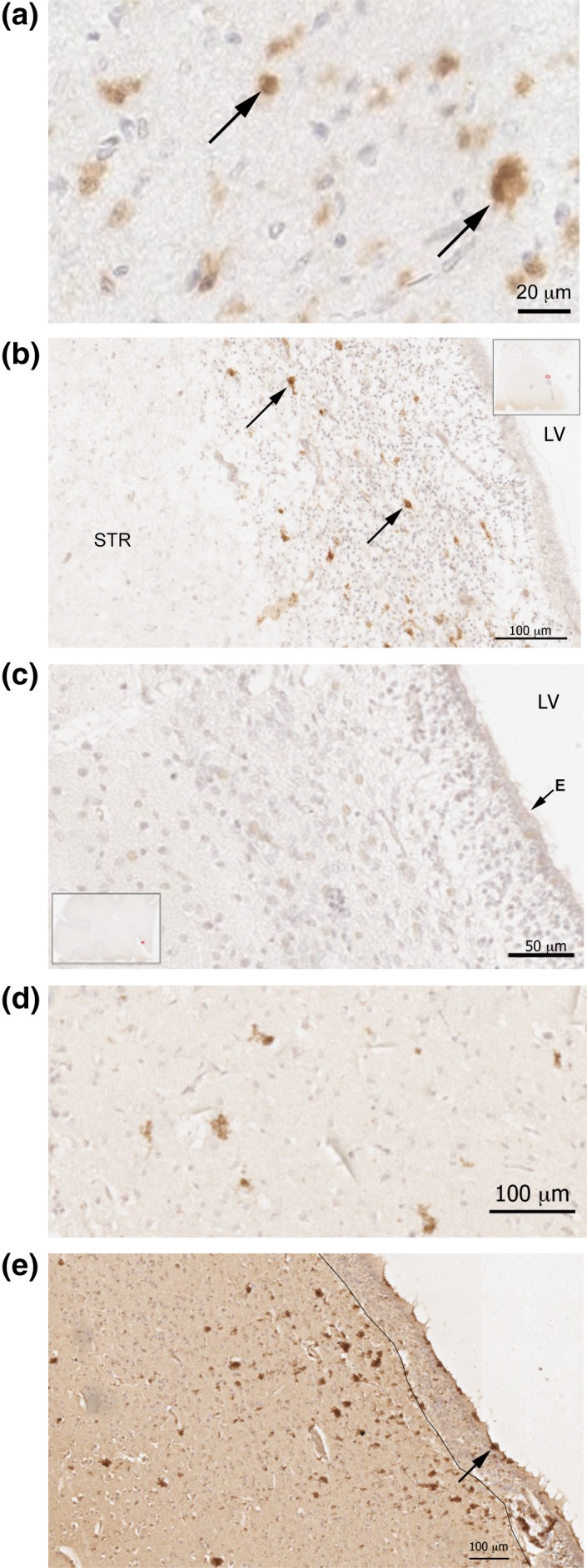
Gal‐3 expression is increased in human perinatal hypoxia/ischaemia. (a) High magnification photomicrograph of Gal‐3 immunopositive cells in the striatum of a patient with subacute H/I. Arrows point to two Gal‐3+ cells. (b) Gal‐3 + cells in the SVZ of a patient with acute hypoxia. Typical examples shown with arrows. There is a high density of small glia‐like nuclei in the SVZ and in this section the SVZ is widely expanded. The inset show a low magnification image of the section and the small red outline within it shows the location of b. LV, lateral ventricle. STR, striatum. (c) An example of a section from an individual with minimal hypoxia/ischemia containing no detectable Gal‐3 immunoreactivity. The section includes the ependymal layer (E) of the LV, the SVZ and the striatum. The inset shows the location of the photomicrograph section in (c). (d) High magnification photomicrograph of several Gal‐3+ cells in the striatum of a subacute H/I patient. (e) A chronic H/I patient with strong Gal‐3 immunopositive cells in the ependymal layer (arrow) and in the adjacent SVZ and CN. In this section, the border of the SVZ and CN was distinct and is demarcated with a thin black line. Scale bars are indicated in the panels. Gal‐3, Galectin‐3; SVZ, subventricular zone

Gal‐3 is increased in human brains with a variety of pathologies such as multiple sclerosis (James et al., 2016). We predicted that Gal‐3 would be increased in the striatum and the adjacent SVZ since the lining of the lateral ventricle is susceptible to perinatal damage as in periventricular leukomalacia. We selected *n* = 14 cases with perinatal H/I damage and focused on the lateral ventricle and adjacent striatum. Gal‐3 immunohistochemistry revealed Gal‐3+ cells in the SVZ and CN of most patients with minimal, acute, subacute, and chronic H/I (Figure 6a,b,d,e). Gal‐3+ cells in the striatum and the SVZ were usually found in distinct subregions or clusters and exhibited a variety of morphologies. Gal‐3+ cells were also found in the cerebral cortex of most H/I cases (data not shown). A few cases had negligible Gal‐3 immunoreactivity in the regions examined (Figure 6c) and these provided useful negative controls. Based on neuropathological examination, absence of inflammation was characteristic of cases with minimal or acute H/I, thus these cases were grouped together. Subacute and chronic cases were also similar in the presence of inflammatory response/vascular changes and these were grouped together for quantification. We selected areas in the striatum and SVZ with Gal‐3+ cells and quantified the percent of cells within them that exhibited robust Gal‐3+ immunoreactivity (see Supplementary materials and methods). The results show significant differences between the two groups (minimal/acute H/I vs. subacute/chronic H/I); the subacute/chronic group exhibited significantly more strongly labeled Gal‐3+ cells (6.22 ± 2.30%) compared with the minimal/acute group (0.69 ± 0.02%) (*p* = .04).

## 4 DISCUSSION

The SVZ is a major source of glial cells in the postnatal forebrain, yet the factors that regulate gliogenesis and that balance astrocyte versus oligodendrocyte production are unclear. Our work disentangled Gal‐3 from inflammation‐related effects and revealed that Gal‐3 loss‐of‐function reduces postnatal gliogenesis while increasing Gal‐3 promotes it. Gal‐3 OE was also sufficient to cause a fate shift, it increased astrogenesis and decreased oligodendrogenesis. We found that Gal‐3 binds to the BMPR1α, that it increases BMP signaling, and that the BMPR1α is necessary for the fate shift caused by Gal‐3 OE. We also showed here that Gal‐3 expression is increased in the human perinatal SVZ and striatum in hypoxia/ischemia, further justifying the study of Gal‐3 OE. Together, our data reveal a novel mechanism showing Gal‐3 regulates basal gliogenesis and together with BMP signaling may shape postnatal SVZ glial fate choices.

One of our central questions had been whether Gal‐3 regulates normal development or if it only becomes functionally relevant when it is increased in disease. Gal‐3 knockout mice show few phenotypic changes, suggesting Gal‐3 is dispensable for healthy development and function (Colnot et al., 1998). However, Gal‐3 is ubiquitously increased in brain pathology and regulates various functions including tumorigenesis (Liu & Rabinovich, 2005). Models of stroke and multiple sclerosis in adult Gal‐3 knockout mice demonstrate it is necessary for angiogenesis and chemokine‐induced immune cell infiltration, respectively (Hillis et al., 2016; James et al., 2016; C. C. Young et al., 2014). Inflammation plays pivotal roles in these diseases, causing multiple cellular and molecular changes that make it difficult to interpret Gal‐3's effects. Additionally, Gal‐3 can itself induce inflammation by attracting and activating macrophages during ischemia, demyelination, and obesity (Hoyos et al., 2014; Lalancette‐Hebert et al., 2012; P. Li et al., 2016). Given this background, it was not altogether surprising that Gal‐3 expression was increased in the human brain in hypoxia/ischemia. H/I events and resultant pathologies are quite variable in humans and are thus difficult to study in postmortem sections and in animal models. We found a range of severity in Gal‐3 expression that was lesser in minimal and acute cases but greater overall in subacute and chronic cases.

By not using a disease model, we provide strong evidence that Gal‐3 OE itself does not cause inflammation in the absence of injury in the postnatal brain. We examined the SVZ and striatum at 3, 7, and 17 days after Gal‐3 OE and showed that CD45+ and Iba1+ cells were unaffected. The former labels all immune cells and the latter is used as a marker of microglia. Neither cell density nor activation state was altered after Gal‐3 OE. We also showed that Gal‐3 OE and knockdown did not increase the rate of apoptosis, which was an important control as cell death can occur during inflammation. Although the expression of various inflammatory cytokines may have been altered by Gal‐3 in this study, based on the data in aggregate, we believe inflammation was not induced. Thus, we studied Gal‐3 function in the absence of inflammation allowing us to uniquely assess its specific effects when increased in expression.

The postnatal SVZ is gliogenic (Levison et al., 1993; Levison & Goldman, 1993), astrogenesis predominates in the first week and is gradually overtaken by oligodendrogenesis (Sauvageot & Stiles, 2002). Our data reveal postnatal Gal‐3 as being instrumental in overall plSVZ gliogenesis; plSVZ‐derived striatal gliogenesis was reduced after Gal‐3 down‐regulation and conditional knockout and increased after Gal‐3 overexpression. These data show that Gal‐3 is instrumental in regulating rates of SVZ‐derived gliogenesis in the striatum. Our study was well within the second half of the gliogenic period, we electroporated at P2 and evaluated at P4 and later time points. In rodents, the onset of astrogenesis is E18 with peak astrocyte progenitor proliferation during the early postnatal period (Miller & Gauthier, 2007). Therefore, electroporating Gal‐3 constructs at E18 may provide additional insight into Gal‐3's effects on gliogenesis, and when Gal‐3 signaling is most important in gliogenesis.

Gal‐3 gain‐of‐function did not just generically increase gliogenesis but specifically increased astrogenesis by 17DPE. Gal‐3 OE also increased the labeling index of SVZ GFAP+ cells that expressed Ki67 at 7DPE. We do not know the exact identity of these GFAP+Ki67+ cells, as they could be mitotic NSCs or glial progenitors. However, Gal‐3 OE did not increase the percent of GFAP+BrdU+ cells (label‐retaining cells) or GFAP+BrdU+EdU+ (mitotically active label‐retaining cells) suggesting Gal‐3 OE did not alter BrdU label‐retaining NSCs or their cell‐cycle re‐entry. SVZ GFAP+ cells that exhibited increased Ki67 expression upon Gal‐3 OE may have been astroglial precursors or even niche astrocytes, but the latter are not typically mitotic (Doetsch, Caille, Lim, Garcia‐Verdugo, & Alvarez‐Buylla, 1999). Interestingly, we also showed that Gal‐3 OE increased the percent of SVZ GFAP+ cells that express Phi3. This suggests that rather than the increase in Ki67 labeling index possibly being due to lengthened cell cycle time, it is due to increased numbers of proliferating SVZ glial precursor cells. Moreover, SVZ‐derived striatal astrocytes exhibited a more mature phenotype after Gal‐3 OE, displaying reduced expression of the immature astrocyte marker vimentin and increased expression of the mature markers S100β and GFAP. This is compatible with a scenario where Gal‐3 OE increases astrocyte maturation. At P19 the predominant glial cell type generated are oligodendrocytes, but Gal‐3 OE reversed this and reduced oligodendrogenesis, suggesting it also affects glial fate choices.

An alternative explanation for the altered astrocyte to oligodendrocyte ratio after overexpression is that Gal‐3 induced cell death. Increased cell death of oligodendrocytes and/or decreased cell death of astrocytes could have contributed to the increased ratio of astrocytes to oligodendrocytes. However, we showed that Gal‐3 OE did not significantly alter the number of cells going through apoptosis (A.Caspase‐3+) at 7 or 17 DPE. As well, it is difficult to characterize which cell type is undergoing apoptosis as marker proteins generally degrade while cells are dying. We did not find any evidence of A.Caspase‐3+ and Olig2 colocalization in the SVZ at 3 DPE (data not shown). Other types of cell death such as ferroptosis or necroptosis may also have affected our cell numbers, but these are rare events in homeostasis and normal development and we would expect baseline levels of these types of cell death to be very low. Investigating other modalities of cell death in the pSVZ is largely unexplored and was beyond the scope of this study.

The subregional differences of the SVZ niche are fascinating and important in terms of embryonic origins and cell subtype generation (K. M. Young, Fogarty, Kessaris, & Richardson, 2007). There is excellent evidence from the Raineteau group that Wnt signaling, which can influence gliogenesis, is restricted to the dorsal SVZ (Azim, Fischer, et al., 2014; Azim, Rivera, Raineteau, & Butt, 2014). As well, the Doetsch group have recently shown with single cell RNAseq that the septal SVZ may be primarily gliogenic (Mizrak et al., 2019). Therefore, in future work it will be important to determine if Gal‐3 regulates gliogenesis in other SVZ subregions.

BMP exposure increases astrogenesis and astrocytic fate/differentiation (Gross et al., 1996; Mabie et al., 1997). Therefore, the glial fate‐shift induced by Gal‐3 OE suggested that BMP signaling was involved. Multiple experimental approaches demonstrated that indeed this was the case. We showed that Gal‐3 increased BMP signaling and it is important to note that Gal‐3's effects on BMP signaling preceded the striatal fate shift, suggesting causation. Importantly, reducing BMP signaling via BMPR1α conditional knockout blocked the Gal‐3 OE‐induced shift from oligodendrogenesis to astrogenesis. This shows Gal‐3's effects are largely dependent on BMPR1α signaling. However, the Gal‐3 OE‐induced GFAP upregulation was only partially reversed after BMPR1α loss, suggesting it may regulate GFAP expression in a BMP‐independent pathway.

Postnatal electroporation results in labeling of cells in the expected temporal lineage progression in the SVZ (Doetsch et al., 1999; Garcia, Doan, Imura, Bush, & Sofroniew, 2004). Electroporation initially targets the radial glia‐like stem cells lining the lateral ventricles (Barnabe‐Heider et al., 2008; Boutin et al., 2008; Chesler et al., 2008; Sun et al., 2018). Within a few days, as the stem cells divide, labeled transit‐amplifying progenitor daughter cells appear. By approximately a week post electroporation, lineage‐specific progenitors—neuroblasts or glioblasts are labeled in the SVZ. Thereafter, labeled neuroblasts and glioblasts can be observed migrating to their target destinations in the OB, striatum and forebrain (Barnabe‐Heider et al., 2008; Boutin et al., 2008; Chesler et al., 2008; Sun et al., 2018). Based on our analysis of Gal‐3 knockdown and conditional knockout, loss of Gal‐3 occurred within 3 days and lasted at least 17DPE. Similarly, Gal‐3 OE occurred at all these time points. We do not know precisely at what stage of lineage progression Gal‐3 regulates gliogenesis. However, we know that BMP signaling is upregulated following Gal‐3 overexpression as early as 2 days (BRE‐luciferase in vitro) and 3 days (pSMAD in vivo). This is followed by an increase in the Ki67 as well as Phi3 labeling indices of SVZ GFAP+ cells at 7DPE, culminating in the gliogenic effect observed at 17DPE. Future work using cell stage specific Cre driver lines may help elucidate when during SVZ lineage progression Gal‐3 mediated BMP signaling regulates gliogenesis.

BMP signaling mediates NSC quiescence and suppresses proliferation (Mercier & Douet, 2014; Mira et al., 2010). We thus expected Gal‐3 to affect NSC self‐renewal. However, Gal‐3 OE did not alter pSVZ NSC proportions, self‐renewal, activation, or olfactory bulb neurogenesis. Gal‐3 mediates Notch signaling in osteoblasts (Nakajima et al., 2014) and may have stimulated Notch signaling to increase NSC maintenance and counterbalance its effects on BMP signaling. Alternatively, one can envisage a scenario where the SVZ harbors Gal‐3 responsive and Gal‐3 nonresponsive cell populations. Gal‐3 may have caused terminal differentiation of Gal‐3‐responsive cells and thereby affected a subpopulation of pSVZ cells, while the remaining Gal‐3 nonresponsive cells compensated for any shrinkage of the NSC pool. Supporting this, Gal‐3 affected BMP signaling in a subset of SVZ cells. Since neither NSC dynamics nor OB neurogenesis were influenced by Gal‐3 OE, we propose Gal‐3 responsive cells are glial progenitors. Indeed, Gal‐3 OE changes were restricted to the glial lineage.

We propose that the mechanism whereby Gal‐3 increases BMP signaling and regulates gliogenic fate choices involves direct interaction between Gal‐3 and BMPR1α. There was evidence that BMPR1α and BMPR2 are glycosylated and bind Gal‐3 in other systems (Hirschhorn, Levi‐Hofman, Danziger, Smorodinsky, & Ehrlich, 2017; Zhang et al., 2017). Interestingly, loss of BMPR1α glycosylation reduced its expression on the cell membrane and decreased Smad1/5/8 phosphorylation (Hirschhorn et al., 2017). We found that BMPR1α is necessary for the Gal‐3 OE effects and confirmed that Gal‐3 binds to this receptor. The downstream effect of increased pSmad1/5/8 is a hallmark of BMP signaling. Gal‐3 can bind to a variety of glycosylated receptors such as the EGFR and transforming growth factor beta (TGFBR) receptors, delaying their endocytosis and prolonging their activation (Partridge et al., 2004; Zhang et al., 2017). We postulate that a similar mechanism may prolong and enhance BMPR1α signaling, but further studies are needed to validate that idea. Alternative mechanisms include signaling via tyrosine kinase receptors like EGFR and activation of the MAPK/ERK pathways. Gal‐3 does not bind to EGFR in adult SVZ cells but loss of Gal‐3 function increased the relative levels of pEGFR (Comte et al., 2011). Therefore, it is also possible that Gal‐3 affects alternative signaling pathways in plSVZ cells such as EGFR‐driven MAPK/ERK. Future biochemical studies will be necessary to determine if these pathways are activated by Gal‐3 in the SVZ.

We showed in this study that Gal‐3 is necessary and sufficient for plSVZ gliogenesis, is increased in human H/I, but has inflammation‐independent functions in the healthy murine brain. Without eliciting inflammation, augmenting Gal‐3 levels increased gliogenesis whereas decreasing it reduced gliogenesis. Furthermore, increasing Gal‐3 expression induced astrocytic fate and differentiation, and simultaneously suppressed oligodendrogenesis; an effect dependent on BMP signaling. We also demonstrated that Gal‐3 interacts with BMPR1α and increases BMP signaling in the plSVZ. The developmental effects and molecular mechanisms uncovered contribute to our understanding of normal gliogenesis and have important implications for disease.

## AUTHOR CONTRIBUTIONS

F.G.S and O.A conceptualized the study; O.A., L.C.S., J.N., S.D., M.M., I.A., T.T., and V.M.L. performed experiments; O.A., E.O., and F.G.S. drafted and wrote the article. All authors approved the final version of this manuscript.

## CONFLICT OF INTERESTS

All authors have no conflict of interests to disclose.

## Supporting information


**Appendix S1:** Supporting Information

**Supplementary Figure S1**

**Supplementary Figure S2**

**Supplementary Figure S3**

**Supplementary Figure S4**

**Supplementary Figure S5**

**Supplementary Figure S6**

**Supplementary Figure S7**

## Data Availability

The data that support the findings of this study are available from the corresponding author FS upon reasonable request.
