# Supplementary material for: Galectin‐3 modulates postnatal subventricular zone gliogenesis
Source: Glia. 2019 Oct 18;68(2):435–50. doi: 10.1002/glia.23730 (PMC6916335; doi:10.1002/glia.23730)
Supplement: Supplementary file 1 — Appendix S1: Supporting Information [file GLIA-68-435-s001.docx]

**Supplementary materials and methods**

**Supplementary Table 2. Primer sequences used for genotyping Floxed Gal-3 mice**

|  | Primer | Sequence | Amplicon |
| --- | --- | --- | --- |
|  | Common | 5′-TGGGGATGTCTACCACACCT-3′ |  |
|  | Gal-3^+^ | 5′-CCCATTTCAACCCATAATTCA-3′ | 284bp |
|  | Gal-3^fl^ | 5′-GAACTTCGGAATAGGAACTTCG-3′ | 146bp |

**Human sections image acquisition and quantification**

Gal-3+ cells (DAB channel) were counted using “positive cell detection” default parameters with detection image set to "optical density sum", median filter radius set to 1 millimeter, minimum area set at 30 millimeter^2^, split by shape set to false, score component set to Cell:DAB OD max, threshold +1 to 0.05, threshold +2 to 0.28, threshold +3 to 0.4, and single threshold set to false. Only cells with threshold +3 were used for statistical analysis to ensure we were quantifying robust Gal-3+ cells.

**β-galactosidase staining**

8 week-old Gal-3^fl/fl^ mice were anesthetized with pentobarbital (150 mg/kg, IP) and perfused transcardially with 4% paraformaldehyde (PFA). The brains were removed, fixed in 4% PFA for 24 hours, cryoprotected in 30% sucrose and frozen. Brain sections of 30 μm thickness were cut on a chilled microtome and used for β-galactosidase staining. The sections were washed with PBS for ten minutes and with detergent rinse (0.02% Igepal, 0.01% Sodium Deoxycholate and 2 mM MgCl2 in 0.1 M phosphate buffer) for another ten minutes. After that the sections were immersed in X-Gal Staining Solution (0.02% Igepal, 0.01% Sodium Deoxycholate, 5 mM Potassium Ferricyanide, 5 mM Potassium Ferrocyanide and 2 mM MgCl^2^ diluted in 0.1 M Phosphate buffer, with 1 mg/mL of X-Gal (Sigma)) overnight at 37^0^C in the dark. The next day the sections were post-fixed in 4% PFA for ten minutes, rinsed in PBS and washed for ten minutes with PBS. Then they were washed with distilled water twice for ten minutes and counter-stained with Nuclear Fast Red (Sigma). The sections were rinsed in distilled water, and washed in distilled water for two minutes. The slides were mounted with FluorSave and coverslipped.

**Nucleofection and Luciferase Assay**

1-4 X 10^6^ NSC/NPC cells from tertiary neurospheres were nucleofected with 5 μg DNA/Plasmid/condition using an Amaxa™ Biosystems Nucleofector I as per the Amaxa™ Mouse Neural Stem Cell Nucleofector™ Kit instructions. For luciferase assays, 1 μg of pGL3 BRE Luciferase and pGL4.75 [hRluc/CMV] (20:1) along with 5 μg of pCAG-Gal-3, shGal-3, or shNT plasmids were used for nucleofection. Cells were then cultured for 2 days before lysates were used to measure Firefly and Renilla luciferase activities with the Dual-Luciferase® Reporter Assay System (Promega) on a Dynex luminometer, according to the manufacturer manual.

**Neurosphere generation and culture**

Hypothermia was induced in P4 mice, followed by decapitation, brain extraction and SVZ dissection. Brains were washed in Hank’s Balanced Salt solution (HBSS) before coronal sectioning into 500 μm slices on a McIlwan tissue chopper. The lateral wall of the lateral ventricle was microdissected under a Leica MZ12 dissecting microscope. SVZ tissue was dissociated by incubation in Accutase® (Sigma) for 10 min at 37°C, followed by trituration using a p1000 Pipetteman® and declumped by passing through a 40 μm cell strainer (BD Falcon). Next, we washed cells 2 x in Neurobasal A+ (NB-A+) (Neurobasal-A medium + 1% B-27 + 1 x GlutaMax + Penicillin/Streptomycin 1 x; (Gibco)). The cell pellet was then suspended in NB-A+ and cells counted using a haemocytometer and/or Scepter™ 2.0 (Millipore), and viability was quantified using Trypan blue (Flukar) exclusion. After the final wash, SVZ cells were diluted to 100 cells/μl in growth medium (NB-A+ GF) (NB-A+ supplemented with 20 ng/ml EGF (Sigma E9644) and 20 ng/ml FGF-2 (R&D Systems 233-FB) and seeded into non-tissue-culture-treated 6 wells plates (BD Falcon) cultured in a 37°C-5% CO_2_ incubator. For culture at clonal density, cells were diluted to 10 cells/μl and cultured in Costar® 96-well plates for 7 days before passaging or analysis.

**Morphological classification of glial cells**

Imaging for morphological classification of striatal GFP+ cells was done using a Zeiss Plan-Apochromat 40x/1.4 oil DIC M27 objective. We binned cells into three categories: astrocytes, oligodendrocytes, or undetermined, based on established morphological criteria (Stensaas and Stensaas, 1968a, b; Levison and Goldman, 1993; Butt *et al.*, 1994; Ramón y Cajal, 1995; Ogata and Kosaka, 2002). Cells were classified as astrocytes if they satisfied 3 or more of the following: highly branched secondary and tertiary process (bushy appearance), tertiary processes surrounding parenchymal cells, contact with blood vessels, process contacting the SVZ, non-overlapping astrocyte domains, elongated cell body, or relatively thick primary processes. Cells were classified as oligodendrocytes if they satisfied 3 or more of the following: round cell body, sparsely branching secondary and tertiary processes, processes branching from a single thick primary process, parallel orientation of processes, branching into striatal white matter, or possible crossing of processes from neighboring cells. Undetermined cells failed to express astrocyte or oligodendrocyte specific criteria or expressed a combination of both.

**cDNA generation and quantitative PCR**

RNA was extracted from tissue or cell pellets using RNeasy minikit (Qiagen) according to the manufacturer’s manual. cDNA was synthesised from RNA using High Capacity RNA-to-cDNA Kits (Thermofisher) as per supplier’s instructions. For qPCR, we used Taqman gene expression assays for Lgals3 and Id1, as well as the house keeping genes B2M, ATPAF2, and Actnb. Amplification efficiencies for each assay was calculated based on the standard curve method, and differences between conditions were assessed using the delta-delta Ct method corrected for amplification efficiency.

### **Supplementary results**

### **NSC activation in vivo is not affected by Gal-3 overexpression.**

## To assess if Gal-3 alters SVZ proliferation control or Gal-3 OE electroporated animals were injected with BrdU for 2 days, a pulse of EdU was given 5 days later and 2 hours later brains were harvested (Fig. S5A). BrdU-retaining cells represented a subset of label-retaining cells NSCs and their direct progeny that remained in the SVZ whilst BrdU+EdU+ cells likely represented activated NSCs exhibiting cell cycle re-entry (Fig. S5B). Gal-3 OE did not alter the proportion of labeled cells that were EdU+, BrdU+, or BrdU+EdU+ (Fig. S5C-E). This indicated that Gal-3 did not affect overall SVZ proliferation, the number of label-retaining cells or cell cycle re-entry. Together these data support the finding that Gal-3 OE did not alter the number of activated SVZ label-retaining cells in vivo.

SVZ GFAP expression increases between P0 and P21 suggesting astrocytic maturation (Peretto *et al.*, 2005). Gal-3 is necessary for reactive astrocyte proliferation after injury (Sirko *et al.*, 2015). Our findings showed that striatal astrogenesis is increased after Gal-3 OE. Thus, we hypothesized that Gal-3 promotes astrocytic precursor proliferation in the pSVZ. We used GFAP and Ki67 to label a putative subset of astrocyte precursors (Fig. S5F). Consistent with (Fig. S5C), the percent of labeled SVZ cells that were Ki67+ was not altered by Gal-3 OE 7DPE (Fig. S5G) and 17DPE (Fig. S4C). Moreover, Gal-3 OE did not alter the proportion of GFP+ cells that were GFAP+ in the SVZ 7DPE (Fig. S5H) or 17DPE (Fig. S4D). Interestingly, the proportion of GFP+ GFAP+ cells that were also Ki67+ was significantly increased by Gal-3 OE 7DPE (Fig. S5I), but not at 17DPE (Fig. S4E). These data indicate that while Gal-3 OE did not alter overall SVZ proliferation 7 and 17DPE, it increased the proliferation of a subset of SVZ GFAP+ cells 7DPE, prior to the observed increase in striatal gliogenesis at 17DPE. We posit that these GFAP+Ki67+ cells are putative astrocyte precursors.

### **Olfactory bulb neurogenesis is not affected by Gal-3 overexpression.**

To investigate whether Gal-3 OE influences OB neurogenesis, we examined sections from 17DPE control and Gal-3 OE mice. We used the proportion of GFP+ labeled cells expressing NeuN as a readout for neuronal differentiation (Fig. S6A). We found no significant difference in the number of GFP+ OB cells with neuronal morphology (Fig. S6B) or in the proportion of GFP+ cells that were NeuN+ between controls and Gal-3 OE (Fig. S6C). Moreover, Gal-3 OE did not influence the distribution of GFP+ or GFP+NeuN+ neurons in the OB layers (Fig. S6D-E). Hence, we concluded that Gal-3 overexpression between P2 and P19 did not change OB neurogenesis.

### **Gal-3 affects glial fate choice.**

In addition to GFAP, S100β, and Olig2 we examined expression of Vimentin, a marker of young astrocytes. Fewer electroporated GFP+ cells in the striatum were positive for Vimentin 17DPE of Gal-3 OE constructs (Fig. S7A). This shows that Gal-3 OE increased striatal astrocytic maturation.

### **Gal-3 overexpression increases Smad phosphorylation.**

To determine if the increase in BMP activity was due to increased Smad protein content or phosphorylation, we treated neurospheres nucleofected with control, Gal-3 OE, or shGal-3 plasmids, and with vehicle or 5 ng/ml BMP4 for 24 hrs (Fig. S7E). The cells were harvested for protein 1 day later (2 days post nucleofection). We immunoblotted for pSmad1/5/8, total Smads 1, 4 and 5, Gal-3, and β-actin (Fig. S7E). Neither Gal-3 knockdown nor overexpression altered Smad 4, or 5 expression. Gal-3 OE did not significantly alter pSmad1/5/8 in control samples, but it did so after BMP treatment (Fig. S7D,E).

**Supplementary Figure Legends**

**Suppl. Fig. 1. Galectin-3 expression in postnatal subventricular zone cells.**

A-C: Confocal images of Galectin-3 (Gal-3; white), and GFAP (A), Mash1 (B), or Dcx (C) (red) in the subventricular zone (SVZ). DAPI+ nuclei (blue) and β-catenin immunohistochemistry (green). β-catenin was used to delineate cell membranes. Boxed areas in B and C are enlarged in B’ and C’. A: The arrow points to a cell that expresses GFAP and Gal-3. Scale bar 10 μm. B: The white arrow shows a Mash1+Gal-3+ cell, the yellow arrow shows Mash1+Gal-3+ cell. Scale bars: 20 μm for main panels and 10 μm for enlarged panels. C: Dcx+ cells express membrane-associated β-catenin but do not express Gal-3. Scale bars: 20 μm for main panels and 10 μm for enlarged panels.

**Suppl. Fig. 2. Gal-3 construct validation**

A: Western blot analysis of protein samples from HEK293T cells transfected with the plasmid combinations indicated and analysed 3 days after transfection in vitro (3DIV). B: 3DPE of a plasmid expressing mCherry in the cytoplasm plus control shRNA, Gal-3 knockdown (shGal-3V1), or Gal-3 overexpression (Gal-3 OE) plasmids. Representative confocal images showing DAPI (blue), mCherry (red) and Gal-3 (Gal-3; white) in the subventricular zone (SVZ). Note the altered Gal-3 immunofluorescence intensity 3DPE in the 3 groups. All images were acquired using the same camera settings. Scale bars: 10 μm. C: Quantification of B. Gal-3 mean fluorescent intensity (MFI) in electroporated cells was normalised to mCherry MFI in arbitrary units. N=4 mice/group; MFI was averaged from 56 – 205 electroporated cells per animal. D: 17DPE of plasmids expressing tdTomato in the cytoplasm plus control shRNA or Gal-3 knockdown constructs (shGal-3V1), (shGal-3V2) as indicated. Representative confocal z-stack of sections stained for Gal-3. Arrow: tdTomato+Gal-3+ cells (in control) or tdTomato+Gal-3- (shRNA) cells. Scale bar: 5 μm. E: Quantification of D. Percentage of cytoplasmic tdTomato+ cytoplasmic Gal-3+ cells. N=3 (control), N=5 (V1 shGal3), N=4 (V2 shGal3). F: Production of Gal-3tm1a (or Gal-3^fl^) and Gal-3tm1b alleles by homologous and Cre-recombinase recombination. Adapted from IMPC website, 2014. Note: targeting vector not to scale. G: Gel electrophoresis of genotyping PCR products showing different genotypic combinations for knockout and floxed mice. H: Expression of β-galactosidase in the olfactory bulb (OB), rostral migratory stream (RMS), and SVZ of P4 and 8 week old mice in Gal-3^fl/fl^ mice. Insets enlarged on the right. Scale bars: 500 μm main panels and 200 μm insets. I: Quantification of the percent of GFP+ electroporated cells expressing Gal-3 in Gal-3^fl/fl^ mouse SVZ electroporated with control (C) GFP plasmids or with Cre-expressing (Cre) plasmids. J: Two examples of confocal microscopy optical sections showing minimal Gal-3 immunofluorescence (white) in cells electroporated with Cre-recombinase expressing plasmids (GFAP+ (red), GFP+ (green), 17DPE. Scale bar = 10 microns.

**Suppl. Fig. 3. Gal-3 does not induce inflammation in the plSVZ and striatum. (Related to Figure 1 - main text).**

A: Representative projection images of confocal z-stacks of control and Gal-3 OE 7DPE and 17DPE showing DAPI nuclei (blue), GFP (green), and CD45 immunohistochemistry (red). CD45+ microglia were not different after Gal-3 OE at any time point examined. Scale bars: 20 μm. B: Representative confocal images of sections stained for Iba1 and DAPI 3DPE. The SVZ is delineated in white lines. LV: lateral ventricle. Scale bar: 30 μm. C: Quantification of microglial density and activation status (resting, intermediate, activated) in the SVZ and striatum (Str) at 3DPE. ns: non-significant. N=3. D: Iba1+ microglia classified based on morphology. Cells were categorized into resting (small cell body with more than 4 branches), intermediate (amoeboid or round with 3-4 processes) or activated (round with one or two thick processes). Scale bar: 10 μm. E: Quantification of activated caspase-3 (A.casp3)+ cells in OB of control and Gal-3 OE 7DPE.

**Suppl. Fig. 4. Gal-3 OE does not alter SVZ proliferation 17DPE.**

A: 17DPE of Gal-3 OE plasmids. Representative confocal image for GFP+ (green), GFAP+ (white), Ki67+ (red) cells and DAPI+ (blue) nuclei of a control animal 17 DPE. The white contour outlines the SVZ, the arrow shows a GFP+GFAP+Ki67+ cell, and arrowhead shows a GFP+GFAP-Ki67+ cell. The boxed area is magnified in an orthogonal view. Scale bars: 20 μm for main panels and 10 μm for orthogonal view. B: The number of GFP+ cells in the lateral SVZ was not altered after Gal-3 OE compared to controls. C: The percent of GFP+ cells that were Ki67+ was not significantly different between the control and Gal-3 OE groups. D: The percent of GFP+ cells that were GFAP+ did not differ in the two groups. E: The percent of GFAP+GFP+ cells that expressed Ki67+ was not significantly different between the control and Gal-3 OE groups. N=4 mice/group for all quantifications in figure.

**Suppl. Fig. 5. Galectin-3 overexpression does not alter SVZ NSC activation but increases astrocytic proliferation 7DPE.**

A: Plasmids, (E/P) and thymidine analogue injection scheme. B: Representative control confocal orthogonal image showing a triple-labeled cell that is GFP+ (green), EdU+ (red) and BrdU+ (white). LV: lateral ventricle. Scale bar: 20 μm. C-E: Quantifications of B, showing proliferation (C), label-retaining cells (D) and cell cycle re-entry (E). N=3-4 mice/group. F: Confocal images showing control and Gal-3 OE groups at 7DPE. The SVZ is outlined. DAPI+ nuclei (blue), GFAP+ (white), GFP+ (green), and Ki67+ (red) cells are shown. Arrows indicate Ki67+GFAP+GFP+ cells, and arrowheads Ki67-GFAP+GFP+ cells. The boxed areas are shown in orthogonal views. Scale bars: 20 μm main panels, 5 μm for insets. G-I: Quantification of F showing the percent of GFP+ cells that are Ki67+ (G), GFAP+ (H), or GFAP+GFP+ cells that also express Ki67 (I). J: Confocal images of the SVZ showing control and Gal-3 OE groups at 7DPE. DAPI+ nuclei (blue), GFAP+ (white), GFP+ (green), and Ki67+ (red) cells are shown. Arrows indicate a Phi3+GFAP+GFP+ cell. Scale bar: 20 μm. K: The percent of GFAP+GFP+ cells that were Phi3+ increased in the Gal-3 OE mice. L: The percent of all the GFP+ cells that were Phi3+ did not increase in the Gal-3 OE mice. N=3-4 mice/group.

**Suppl. Fig. 6. Galectin-3 overexpression does not alter olfactory bulb neurogenesis 17DPE.**

A: Projection images of confocal z*-*stacks from olfactory bulb (OB) sections, 17DPE of control and Gal-3 OE plasmids. Immunohistochemistry for electroporated GFP+ (green) and NeuN+ (red) cells and DAPI+ nuclei (blue). GFP+NeuN+ (arrows) and GFP+NeuN- (arrowheads) are shown in OB layers demarcated by solid lines. Interrupted line marks the estimated boundary between deep and superficial granule layers (D.Gr and Sp.Gr, respectively). Boxed areas are magnified in insets. RMS, rostral migratory stream, M, mitral layer, EPL, external plexiform layer, PGL, Periglomerular layer. Scale bars: 50 μm for main panels and 20 μm for insets. B-E: Quantification of A. The mean number of GFP+ cells per OB section (B), the percentage of GFP+ cells expressing NeuN (C). The percent distribution of GFP+ and GFP+NeuN+ cells in OB layers is shown in D and E, respectively. N=4 mice/group.

**Suppl. Fig. 7. Fate choice, colocalisation, BMP signaling and morphology**

A: Z-stack showing DAPI+ nuclei (blue), and immunohistochemistry for GFP+ (green) and Vimentin+ (Vim; red) cells. Arrows point to GFP+Vim+ and arrowheads to GFP+Vim- cells. 17DPE. B: Percentage of striatal GFP+ cells that expressed Vim decreased 17DPE with Gal-3 OE plasmid. N=3-4 mice/group. C: Representative confocal image of SVZ cells stained for pSmads1/5 and Gal3. Merged image show the orthogonal view. Arrow indicate pSmad1/5+Gal3+ cell. The SVZ is delineated in white lines. LV: lateral ventricle. Scale bar: 10 μm. D: Western blot 24 hrs after nucleofection with shNT (control), shGal-3, or Gal-3 OE. Cells were treated with control or 5 ng/ml BMP4 for 24 hrs. Immunoblot for pSmad 1/5/8, total Smad 4, total Smad 5, Gal-3, and β-Actin. E: Quantification of pSmad1/5/8 band intensities from (E) normalised to total Smad 1. F: BMP responsive element Firefly luciferase activity increased after Gal-3 OE. Normalised Firefly to Renilla activity (RLU: Relative Luminescence Units) expressed as fold change from control. One-sample t-test. n=4 independent experiments. G: Relative expression of Id1 and Gal-3 from NSCs treated with PBS or 10 ng/ml BMP4 for 48hrs. One sample t-test, n=3-4. **Supplementary references**

Butt AM, Colquhoun K, Tutton M, Berry M. Three-dimensional morphology of astrocytes and oligodendrocytes in the intact mouse optic nerve. J Neurocytol 1994; 23: 469-85.

Levison SW, Goldman JE. Both oligodendrocytes and astrocytes develop from progenitors in the subventricular zone of postnatal rat forebrain. Neuron 1993; 10: 201-12.

Ogata K, Kosaka T. Structural and quantitative analysis of astrocytes in the mouse hippocampus. Neuroscience 2002; 113: 221-33.

Peretto P, Giachino C, Aimar P, Fasolo A, Bonfanti L. Chain formation and glial tube assembly in the shift from neonatal to adult subventricular zone of the rodent forebrain. J Comp Neurol 2005; 487: 407-27.

Ramón y Cajal S. Histology of the nervous system of man and vertebrates. New York

Oxford: New York

Oxford : Oxford University Press; 1995.

Sirko S, Irmler M, Gascon S, Bek S, Schneider S, Dimou L*, et al.* Astrocyte reactivity after brain injury-: The role of galectins 1 and 3. Glia 2015.

Stensaas LJ, Stensaas SS. Astrocytic neuroglial cells, oligodendrocytes and microgliacytes in the spinal cord of the toad. I. Light microscopy. Z Zellforsch Mikrosk Anat 1968a; 84: 473-89.

Stensaas LJ, Stensaas SS. Light microscopy of glial cells in turtles and birds. Z Zellforsch Mikrosk Anat 1968b; 91: 315-40.
