# Supplementary figures and images for: Galectin‐3 modulates postnatal subventricular zone gliogenesis
Source: Glia. 2019 Oct 18;68(2):435–50. doi: 10.1002/glia.23730 (PMC6916335; doi:10.1002/glia.23730)

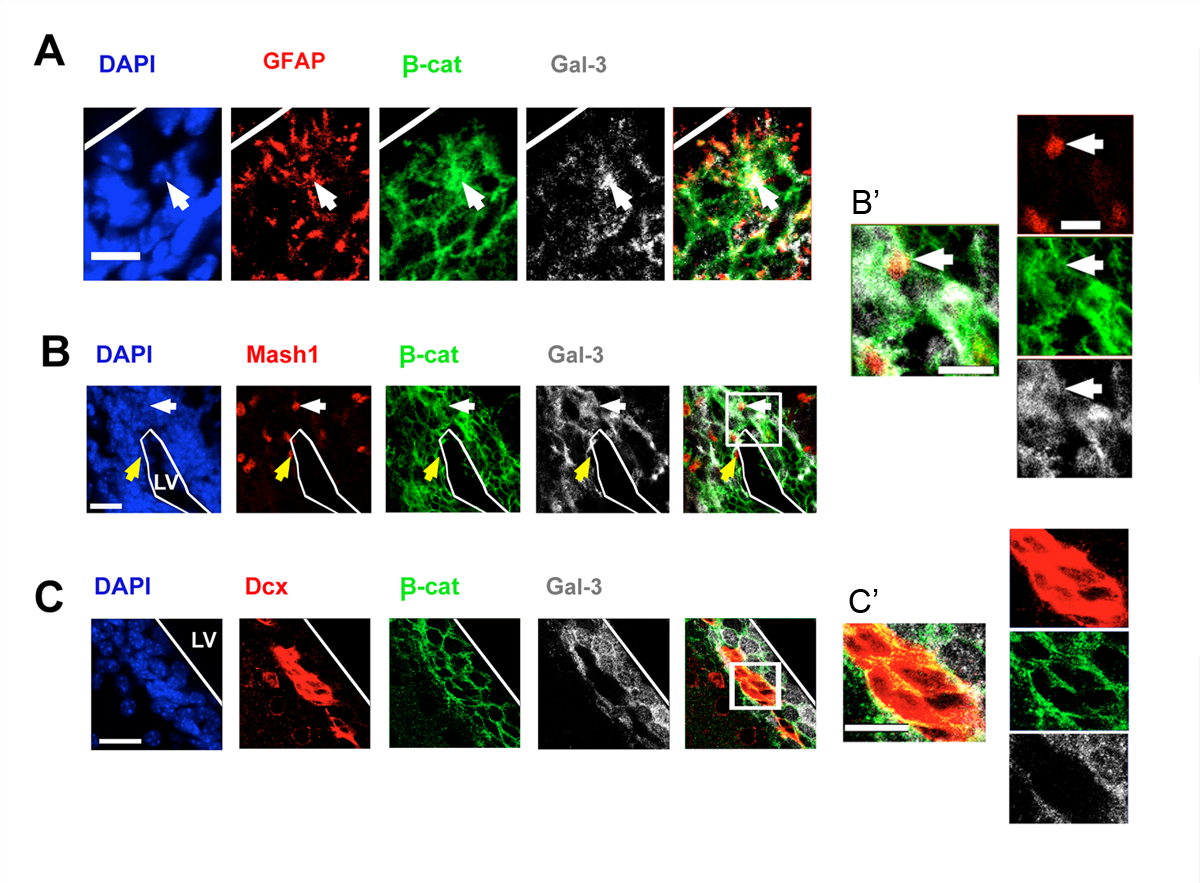

Supplement: Supplementary file 2 — Supplementary Figure S1 [file GLIA-68-435-s002.tiff]

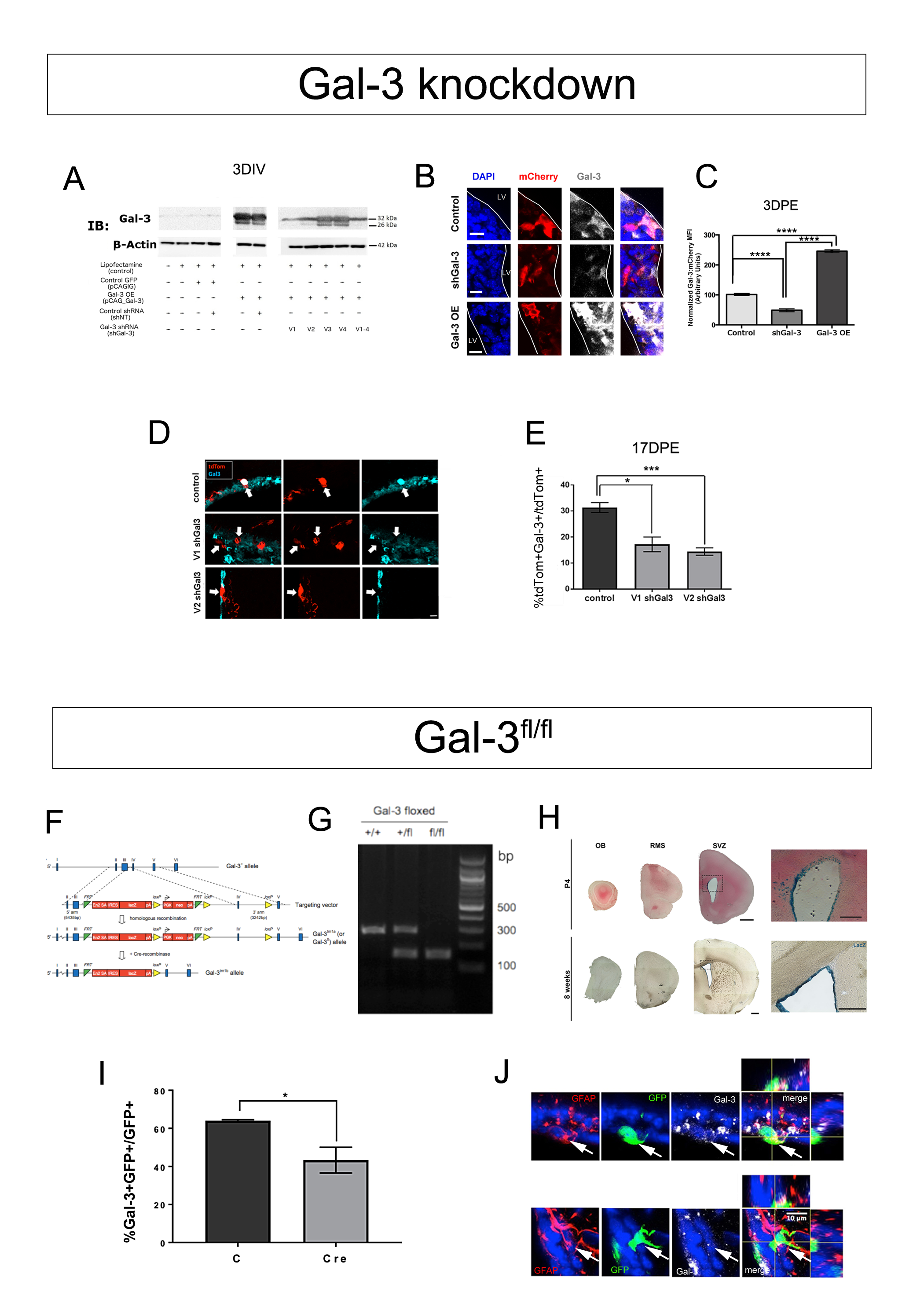

Supplement: Supplementary file 3 — Supplementary Figure S2 [file GLIA-68-435-s003.tiff]

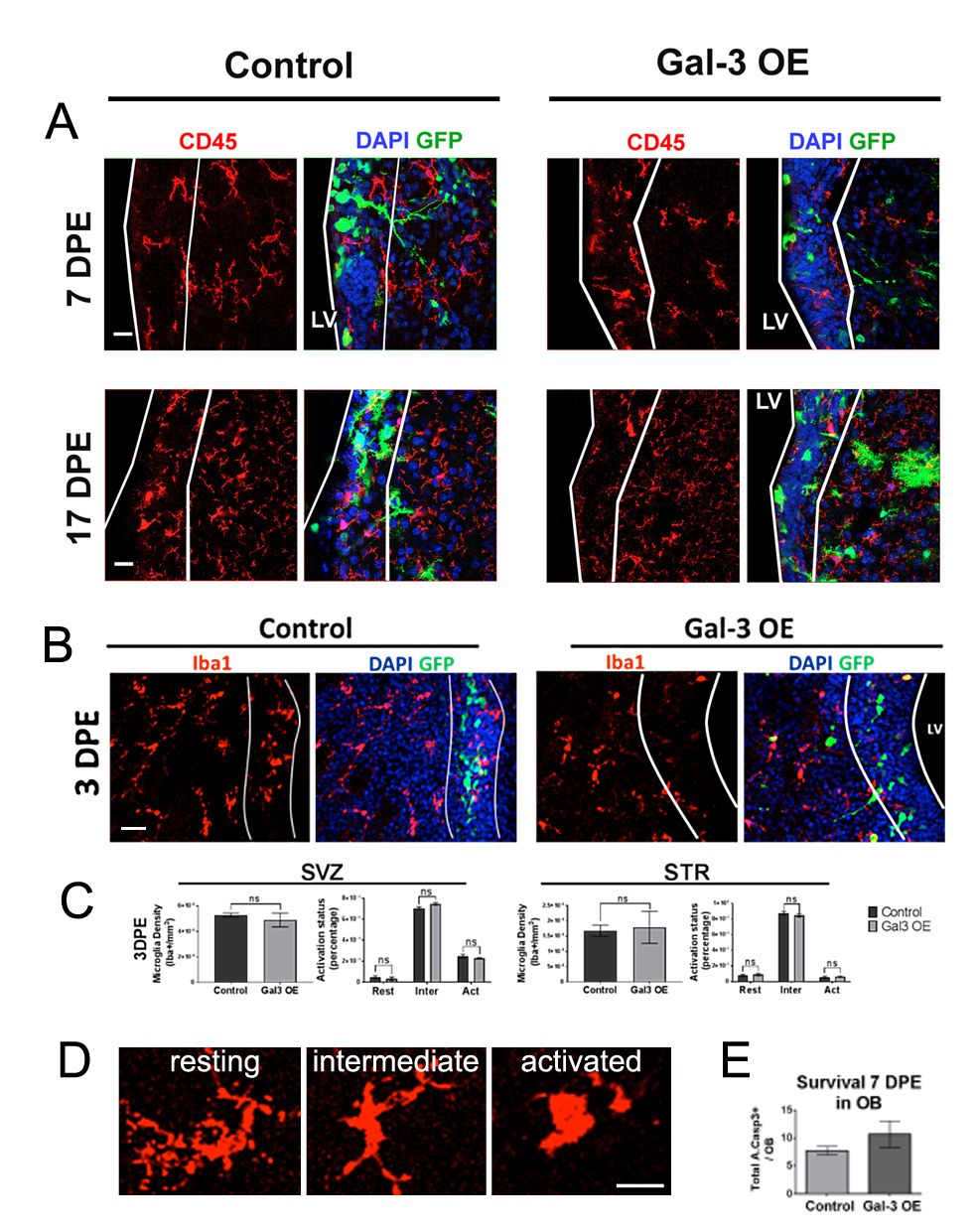

Supplement: Supplementary file 4 — Supplementary Figure S3 [file GLIA-68-435-s004.tiff]

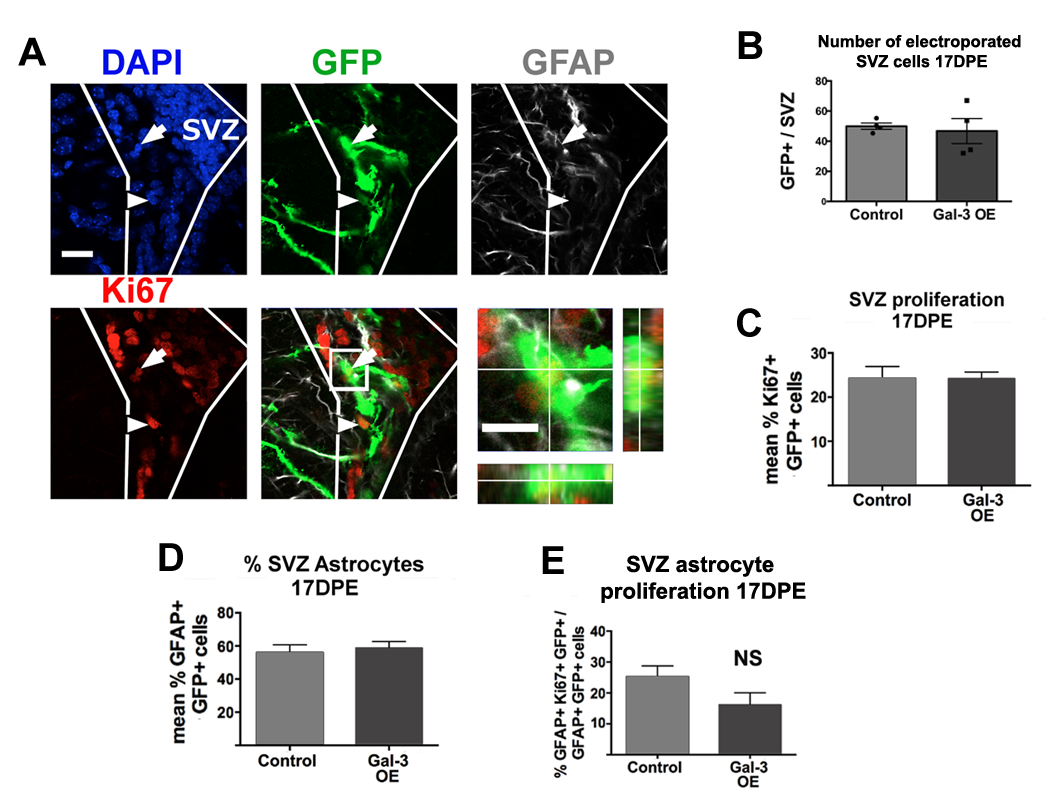

Supplement: Supplementary file 5 — Supplementary Figure S4 [file GLIA-68-435-s005.tiff]

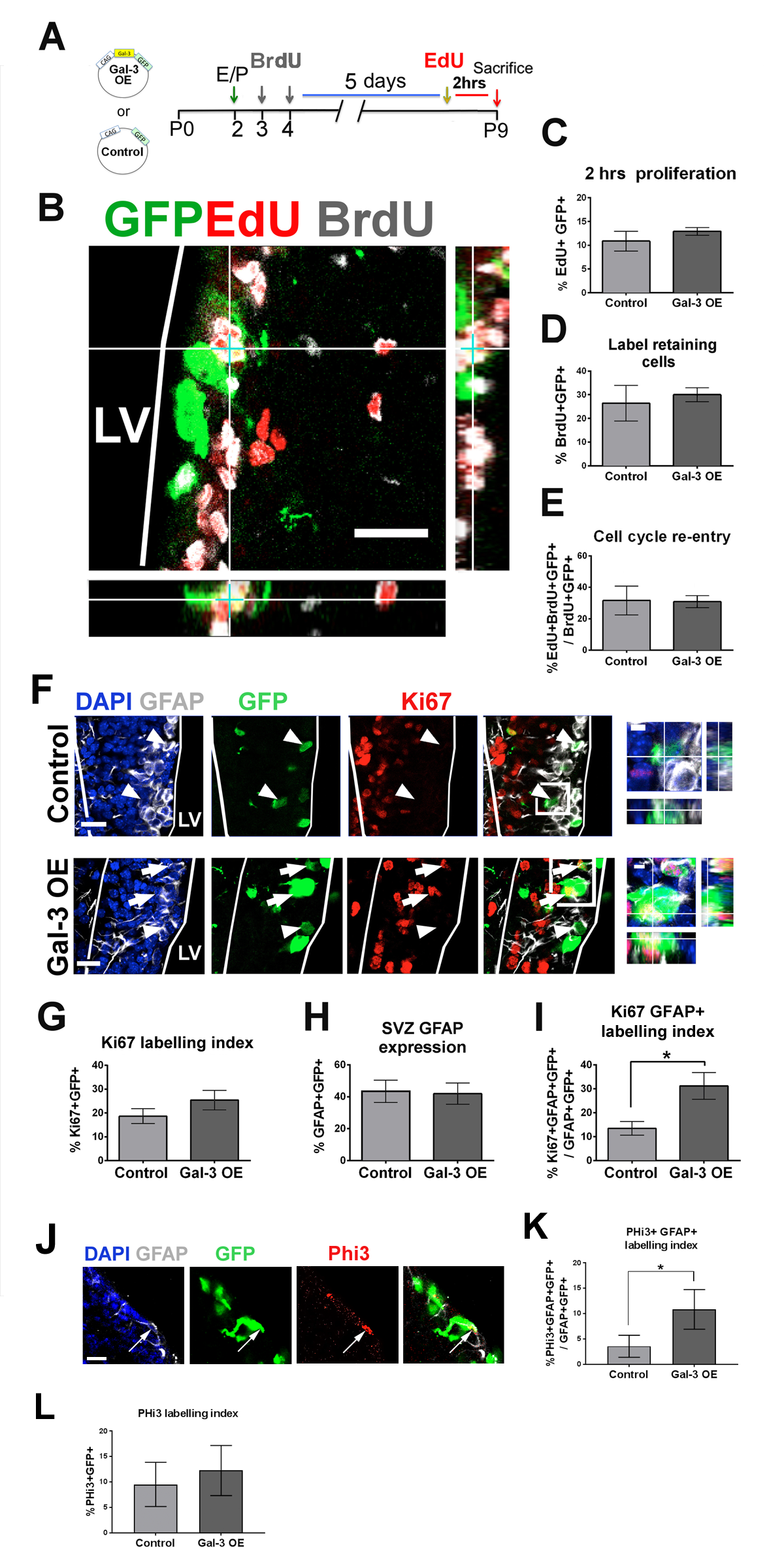

Supplement: Supplementary file 6 — Supplementary Figure S5 [file GLIA-68-435-s006.tiff]

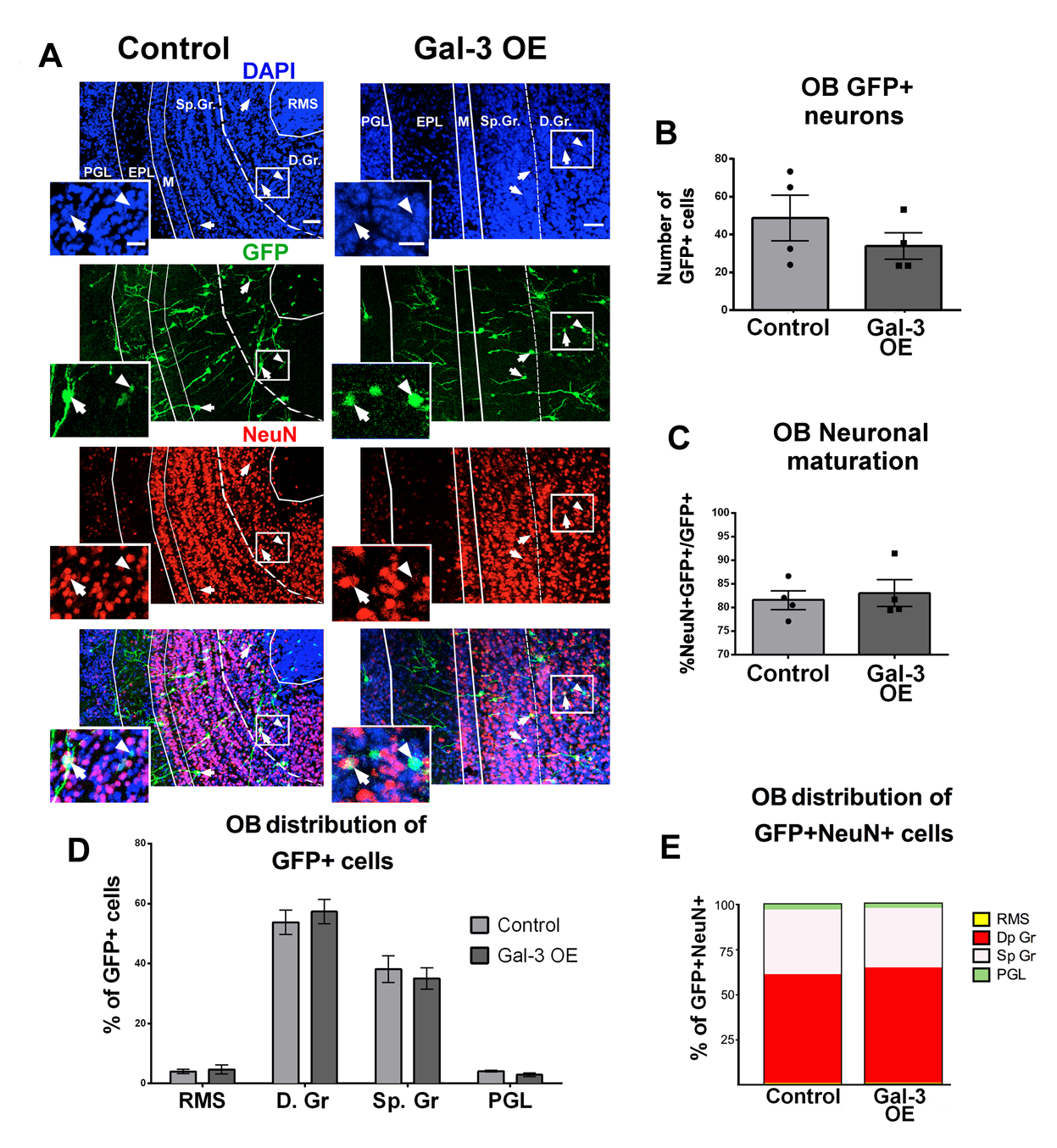

Supplement: Supplementary file 7 — Supplementary Figure S6 [file GLIA-68-435-s007.tiff]

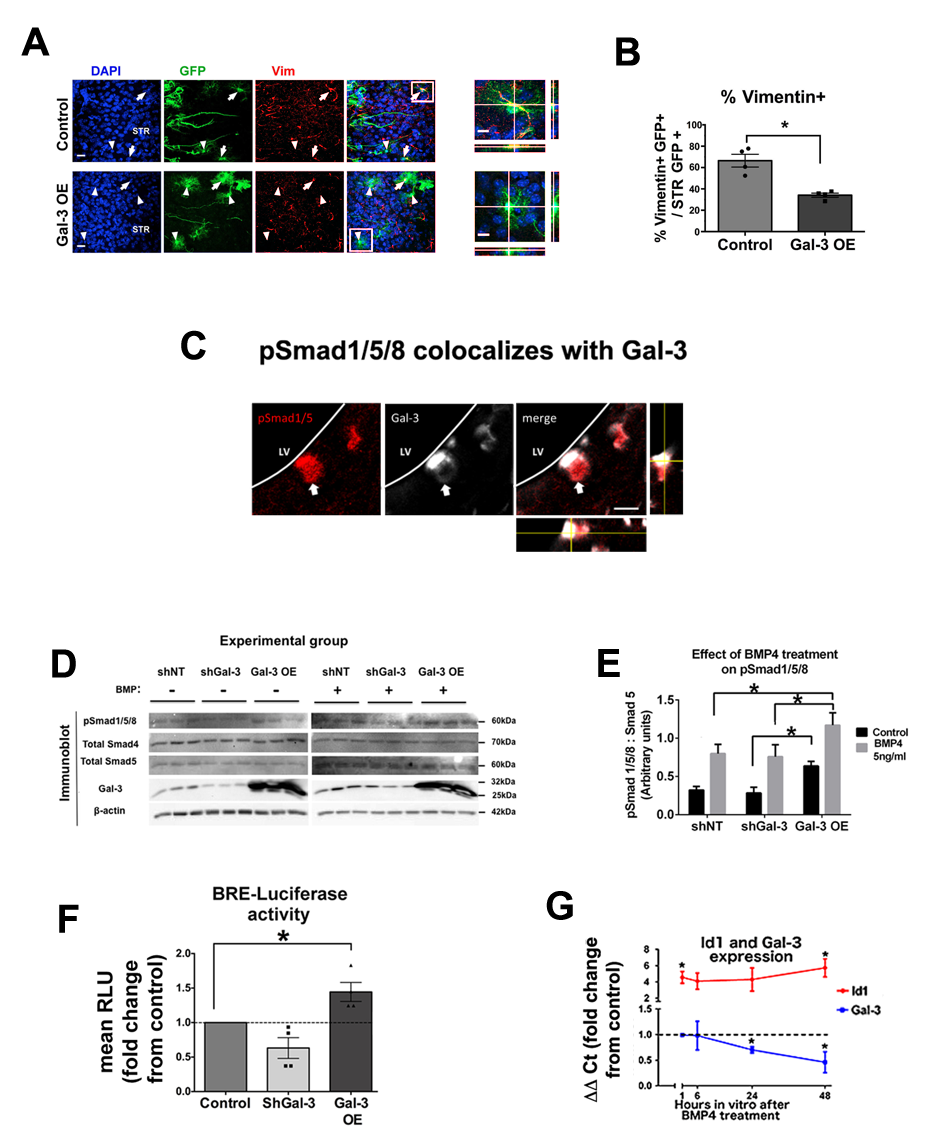

Supplement: Supplementary file 8 — Supplementary Figure S7 [file GLIA-68-435-s008.tiff]
